# Integrative multi-omics and radiomics reveal a TMSB10-driven cell state for non-invasive assessment and precision stratification in breast cancer

**DOI:** 10.3389/fimmu.2026.1794329

**Published:** 2026-04-20

**Authors:** Gui-Xin Wang, Jun-Ming Cao, Cheng-Lu Lu, Yun-Lin Wang, Zi-Yi Chen, Chang-Qing Yang, Shuo Wang, Zhang-Yin Guo, Yue Yu, Shan Cheng, Xin Wang

**Affiliations:** 1 The First Department of Breast Cancer, Tianjin Medical University Cancer Institute and Hospital, National Clinical Research Center for Cancer, Tianjin, China; 2 Key Laboratory of Cancer Prevention and Therapy, Tianjin, China; 3 Tianjin’s Clinical Research Center for Cancer, Tianjin, China; 4 Key Laboratory of Breast Cancer Prevention and Therapy, Ministry of Education, Tianjin Medical University, Tianjin, China; 5 Department of Pathology, Hebei Key Laboratory of Molecular Oncology, Tangshan People’s Hospital, Tangshan, China; 6 Department of Hematology, Tianjin Medical University Cancer Institute and Hospital, Tianjin, China; 7 National Clinical Research Center of Cancer, Tianjin, China; 8 Tianjin Key Laboratory of Cancer Prevention and Therapy, Tianjin, China; 9 Tianjin’s Clinical Research Center of Cancer, Tianjin, China; 10 Department of Thoracic Oncology, Tianjin Lung Cancer Center, Key Laboratory of Cancer Prevention and Therapy, Tianjin’s Clinical Research Center for Cancer, Tianjin Medical University Cancer Institute and Hospital, Tianjin Medical University, Tianjin, China; 11 Respiratory Department, Tianjin Medical University General Hospital, Tianjin, China

**Keywords:** breast cancer, precision medicine, ScRNA-seq, TMSB10, tumor microenvironment

## Abstract

**Background:**

Tumor cell heterogeneity is a fundamental driver of breast cancer aggressiveness, underlying recurrence, metastasis, and therapy resistance. Understanding the biological characteristics and functions of specific tumor cell clusters in the tumor microenvironment is crucial for advancing precision oncology.

**Methods:**

We delineated breast cancer tumor cell heterogeneity by integrating single-cell transcriptomics, spatial transcriptomics, bulk transcriptomics, genomic and radiomic data. The oncogenic functions of the candidate gene *TMSB10* were rigorously validated *in vitro.* To advance individualized patient management, we employed machine learning to develop a non-invasive MRI radiomic model for estimating tumor cluster abundance and a robust prognostic signature for risk stratification.

**Results:**

We discovered a poor-prognosis tumor cell cluster (C1 cluster). C1 cluster exhibited a late evolutionary state, metabolic reprogramming (OXPHOS/glycolysis), and active crosstalk with cancer-associated fibroblasts and endothelial cells. High abundance of C1 cluster was associated with poor survival, specific somatic mutations, and predicted superior response to immune checkpoint blockade, but not to chemo/radiotherapy. The radiomic model based on MRI images was exploratively established for estimating the abundance of C1, and the prognostic model based on C1-derived genetic features significantly stratified the survival risk of breast cancer in multiple cohorts. *In vitro* experiments confirmed that *TMSB10*, a C1 core gene, promotes proliferation, migration, invasion.

**Conclusions:**

This study revealed C1 cluster as a key driver of breast cancer progression and its application for predicting immunotherapy response. Additionally, *TMSB10* was identified as a functional effector of C1 cluster, providing a new and applicable clinical tool for non-invasive detection and prognostic stratification for breast cancer.

## Introduction

As the most frequent tumor in women globally, breast cancer has become one of the main causes of cancer-associated deaths among women (1–3). Unfortunately, the number of deaths from breast cancer worldwide will continue to increase based on current disease trends (4). The high tumor heterogeneity including genetic mutations, complicated tumor microenvironment, and cellular heterogeneity profoundly influences the treatment response, recurrence and metastasis of breast cancer (5, 6). Treatment strategies specified solely by molecular typing are partially limited by the heterogeneity of breast cancer, resulting in different responses to the same treatment even among patients of the same subtype (7, 8). Novel biomarkers and indicators based on the heterogeneity of breast cancer need to be further developed to promote individualized diagnosis and treatment of breast cancer.

Sequencing technologies have rapidly developed over the past two decades, enabling continuous dissection of tumor heterogeneity. Most previous studies focused on tissue-level heterogeneity as revealed by bulk RNA sequencing, such as the PAM50 classification of breast cancer based on transcriptomic profiles of breast cancer tissues (9). However, bulk RNA sequencing is inherently limited in resolving intra-tumoral heterogeneity due to lack of resolution of diversity within the same cell type, intercellular crosstalk among different cell populations, and critical spatial information (10, 11). To address these limitations, the advent of high-resolution spatial and single-cell technologies has revolutionized our ability to dissect intratumoral heterogeneity. Through multi-omics analysis, cellular clonal evolutionary trajectories, key driver molecules, novel cell subtypes, and tumor subtypes are being continually revealed (12–14). For instance, Nalio Ramos et al. (15) identified a subgroup of tissue-resident *FOLR2*^+^ macrophages in breast cancer using single-cell RNA sequencing that help support the functionality of CD8^+^ T cells and exert anti-tumor effects by directly contacting them. Multi-omics analysis by Wu et al. (16) has identified a subtype of antigen-presenting mast cells whose heterogeneous infiltration influences the efficacy of PD-1 blockade therapy. However, these recent studies focused mostly on immune cells and did not touch the most heterogeneous tumor cells. In fact, a number of studies have also reported heterogeneity in breast cancer tumor cells. Liu et al. (17) identified heterogeneity and spatial distribution across six breast cancer tumor cell subpopulations. Another study (18) utilized spatial transcriptomics and single-cell sequencing to reveal changes in glycolysis and oxidative phosphorylation during lymph node metastasis in breast cancer. However, these findings are often hindered by limited cohort sizes and insufficient clinical-translational validation. Systematic delineation of tumor cell heterogeneity and acceleration of translation into clinical practice urgently requires larger multi-platform cohorts and more comprehensive multi-omics analysis.

Here, we integrated spatial transcriptomics, single-cell RNA sequencing, bulk RNA sequencing, radiomics, genomics, machine learning algorithms, and *in vitro* experiments to identify a tumor cluster characterized by high expression of *TMSB10*. We have elucidated both the direct and indirect pro-tumorigenic roles of this cluster within the tumor microenvironment, identified factors associated with its infiltration heterogeneity, and further established an exploratory non-invasive diagnostic model centered on this cluster and a robust cross-platform prognostic model based on its biomarker signature. Furthermore, we found that the infiltration level of this cluster influenced the therapeutic response to immune checkpoint inhibitors, but not to radiotherapy or chemotherapy. The core gene *TMSB10* of this cell cluster was functionally validated in breast cancer cell lines by *in vitro* experiments. These findings provide new insights into the heterogeneity, non-invasive diagnostic assessment, prognostic stratification, and individual therapy of breast cancer, while establishing a new, comprehensive research framework and a theoretical foundation for clinical translation of breast cancer research.

## Methods

**Figure 1:**
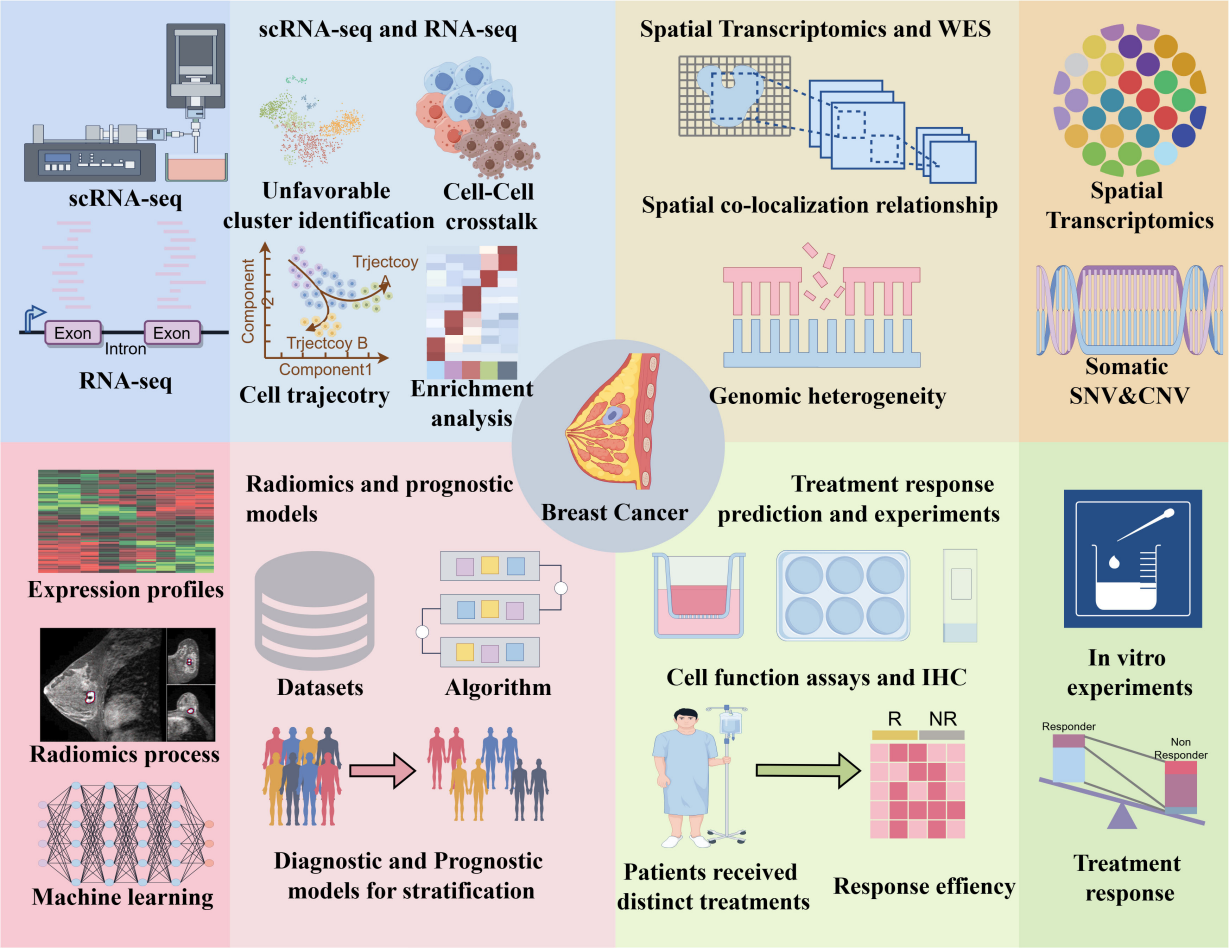
Flowchart of the study. ScRNA-seq, single-cell RNA sequencing; RNA-seq, RNA sequencing; WES, whole exome sequencing; SNV, single nucleotide variants; CNV, copy number variants; IHC, Immunohistochemistry.

The overall study design is illustrated in **Figure 1**.

### Data acquisition

Single-cell RNA-seq data from 31 primary breast cancer samples (GSE180286, GSE176078), spatial transcriptome data (GSE203612), bulk RNA dataset based on microarray (GSE20685, GSE42568, GSE58812, GSE173839) were downloaded from Gene Expression Omnibus (GEO, https://www.ncbi.nlm.nih.gov/geo/). The expression profiles of the TCGA-BRCA cohort were downloaded using the TCGAbiolinks (v2.26) (19) package. In addition, the somatic mutational data, DNA copy number variation data, DNA methylation 450K data, and clinical characteristics of the TCGA-BRCA dataset were directly obtained from the UCSC Xena database (https://xena.ucsc.edu/). The METABRIC cohort was obtained from cBioPortal (https://www.cbioportal.org/). For transcriptome data, samples lacking follow-up information or with lost expression profiles were excluded from subsequent analysis. The information of distinct cohorts was listed in **Supplementary Tables 1**, **S2**.

### Quality control and preprocessing of single-cell data

The DoubletFinder (v2.0.3) (20) package was utilized to eliminate mixed double cells for each sample, with the doublets discovery rate set at 0.8%. Subsequently, the standard single-cell process was executed using the Seurat package. Indicators such as the number of genes detected, the number of transcripts, the proportion of mitochondrial gene expression, and the proportion of hemoglobin gene expression were employed to further filter out low-quality cells. Batch effects from different datasets and among samples within the same dataset were controlled by the Harmony package. The Louvain algorithm was used to distinguish cell clusters based on the similarity between cells, and the number of cell clusters was determined with the assistance of the Clustree (v0.5.0) package. Further, the cell types were annotated by well-established canonical marker genes.

### Downstream analysis of scRNA-seq

The Infercnv package (v1.14.2) (21) was employed to calculate the copy number variation level of epithelial cells, with the expression profiles of normal cells (myeloid cells) used as the reference matrix. The cut-off value was set to 0.1. Epithelial cell clusters with an average CNV score higher than the average CNV score of all epithelial cells were considered tumor cells. The Scissor package (v2.0.0) was used to build the association between the single-cell RNA matrix and phenotype data to identify cells with unfavorable prognosis in primary breast cancer. Specifically, the alpha value was set to 0.02, and the cutoff value was set to 0.2. The scissor+ cells were identified and associated with poor prognosis of breast cancer. Cell preference analysis was conducted to determine the preference of scissor+ cells in each tumor cluster. Differential expression genes among cell types were identified by the FindAllMarkers function. Genes with log2(fold change) > 0.25 and adjusted-*p* value < 10e-5 were considered statistically significant.

Cell developmental analysis was performed using the Vector package. Additionally, cell potential lineage trajectories were inferred by the Slingshot algorithm. Function enrichment analyzes, including GO, KEGG, and Hallmark, were performed using the clusterProfiler (v4.6.2) (22, 23) package. The absolute abundance of the tumor clusters was estimated for each bulk tumor sample using CIBERSORTx in absolute mode, which provides a score that represents the proportion of cells identified as belonging to the distinct clusters within the total transcriptome. Interactions among cell clusters were estimated by the CellChat (v1.6.1) (24) package with default settings. Survival analysis was conducted using the survival package.

### Spatial transcriptome analysis and multimodal intersection analysis

Overall, the Seurat package (v4.3.0) (25–27) was used for the preprocessing of spatial transcriptomes. The spatial transcriptomes matrix was standardized using the SCTransform method, with the resolution determined based on the dimension of principal component analysis. Multimodal intersection analyzes (MIA) were performed to infer the enrichment score of cell abundance in distinct regions. Differential expression genes among distinct regions were identified by the FindAllMarkers function. The differentially expressed genes should meet the following criteria: 1. log2(fold change) > 0.2; 2. Adjusted-*p* value < 0.05; 3. pct.1 > 0.5 & pct.2 < 0.5. Hypergeometric tests were used to detect overlapping relationships between differential genes among spatial regions and cell cluster markers. The GSVA package (v1.46.0) (28) was used to calculate the enrichment score of cell clusters.

### Somatic mutation analysis and copy number variation analysis

Samples were divided into high and low groups according to the median abundance of the C1 cluster. Significant focal somatic mutations were identified and visualized using the maftools package (29, 30), with *p* value evaluated by Fisher’s exact test. Similarly, co-mutated gene pairs and co-rejection gene pairs of the top 10 genes in the high and low groups were also identified by Fisher’s exact test. Additionally, CNV landscapes were analyzed using GISTIC 2.0 (https://www.genepattern.org/), with the confidence level set to 0.99. The top 10 amplified and deleted genomic regions were visualized using the maftools package.

### Potential clinical translation

Samples derived from the METABRIC cohort were divided into high and low groups based on the median value of C1 abundance. Kaplan-Meier curves were used to evaluate the association between the infiltration level of the C1 cluster and distinct treatment types (Chemotherapy, Radiotherapy, Chemotherapy combined with Radiotherapy). Additionally, the immune landscape of the high and low groups was analyzed using multi-scale data from TCGA. The Spearman correlation was used to calculate the mRNA expression and methylation level of immunomodulators. The TIDE and submap algorithms were used to estimate the immune response, with the reference matrix and clinical information of patients receiving CTLA-4 or PD-1 available in the public study (31). Moreover, data from breast cancer patients who participated in the I-SPY2 platform trial were used to further investigate the immunotherapy response rate based on C1 cluster heterogeneity. The Kaplan-Meier plotter and GEPIA2 databases were utilized to analyze the prognostic and immunological role of TMSB10.

### Construction of radiomic model and prognostic model based on machine learning

Magnetic resonance imaging (MRI) data of breast cancer samples were downloaded from The Cancer Imaging Archive (TCIA). Samples with expression profiles and examined by 1.5T GE whole-body MRI systems were included in the subsequent analysis. The tumor area, as the spatial region of interest (3D, volumetric), was manually segmented by two oncologists with five years of experience in breast tumors. Any disagreements were resolved by a more senior doctor. Features of Dynamic Contrast Enhanced-T1 were extracted using 3D slicer software. Samples were randomly divided into the training set and the validation set in a 1:1 ratio. Pearson correlation analysis was utilized to further prioritize valuable features correlated with the abundance of the C1 cluster after Z-score normalization. Finally, LASSO was used to construct the radiomic model to estimate the abundance of the C1 cluster. The discriminative efficiency of the model was assessed using Receiver operating characteristic (ROC) curves.

To further establish a prognostic model for the stratification of breast cancer patients, multiple datasets (TCGA, GSE20685, GSE42568, and GSE58812) were included. GSE20685 was used as the training set, while the others were used as validation sets. The top 50 markers of the C1 cluster were used to construct the prognostic model using the Mime1 package. Over 100 types of machine learning algorithms were employed to build robust models. Publicly published prognostic models of breast cancer were collected and compared with the model we constructed in terms of C-index. Univariate COX analysis was used to evaluate the hazard ratios of candidate genes in multiple datasets. Adherence to the proportional hazard assumption was verified by assessing Schoenfeld residuals. The limma (v3.54.2) (32) package was used to analyze the differentially expressed genes of high- and low- *TMSB10* samples of TCGA based on the median value of *TMSB10* expression level. Subsequently, gene set enrichment analysis (GSEA) was performed to identify the potential downstream signaling pathways using the clusterProfiler package.

### Clinical samples collection

The specimens of breast cancer confirmed by pathology were obtained from Tianjin Medical University Cancer Institute and Hospital. The study was approved by the Ethics Committee of Tianjin Medical University Cancer Institute and Hospital (Ek2021021) and adhered to the ethical guidelines of the Helsinki Declaration. The written informed consent was obtained from the participants.

### Cell culture, plasmids, and transfection

The normal mammary epithelial cell line MCF-10A and breast cancer cell lines MDA-MB-231, BT-549, MCF-7, T47D, BT474, HCC-1954, and CAL-51 were all obtained from ATCC (American Type Culture Collection, Manassas, USA). MCF-10A cells were cultured in MCF-10A-specific growth medium (Procell, China). MDA-MB-231 and MCF-7 were maintained in DMEM medium (Gibco, Grand Island, USA). BT-549, T47D, BT474, HCC-1954, and Cal-51 were maintained in 1640 medium (Gibco, Grand Island, USA). All media were supplemented with 10% fetal bovine serum (FBS, NEWZERUM, Australia) and 1% penicillin/streptomycin (Gibco, Grand Island, USA). Cells were incubated in a humidified incubator at 37 °C with 5% CO_2_. Lentiviral particles were produced by transfecting HEK293T cells with the shRNA targeting TMSB10 (**Supplementary Table 3**) and packaging plasmids (Genomeditech, China) using Lipofectamine 3000 (Invitrogen, California, USA). Viral supernatants were harvested 48 hours after transfection and filtered. Target cells were then infected with the viral supernatants in the presence of polybrene (8 µg/mL). Stable knockdown cells were selected with puromycin (2 µg/mL) for 3 days.

### RNA isolation and quantitative reverse transcription PCR

Total RNA was isolated from cells following the manufacturer’s instructions using the Rapid Cellular RNA Extraction Kit (Sparkjade, China). The concentration and purity of RNA were determined using a NanoDrop 2000 spectrophotometer. For mRNA analysis, the All-in-One First Strand cDNA Synthesis SuperMix for qPCR (Transgene, China) was used for RT-qPCR. TMSB10 and GAPDH primers were synthesized by Genewiz (Tianjin, China), and all specific sequences are listed in the **Supplementary Table 4**.

### Proliferation assay

For the colony formation assay, 1000 cells were seeded into six-well plates and incubated until colonies reached the desired size. Colonies were fixed with 4% paraformaldehyde, stained with 0.1% crystal violet, and subsequently imaged and counted. The EdU assay was conducted using the EdU Assay Kit (RiboBio, China) in accordance with the manufacturer’s instructions. The proportion of EdU-positive cells was quantified and analyzed under a fluorescence microscope (Leica, Germany).

### Migration and invasion assay

Cell invasive capacity of breast cancer cells was evaluated using Matrigel-coated Transwell inserts (BD Biosciences, New Jersey, USA). Briefly, 50,000 cells were seeded into the upper chambers in serum-free medium, while the lower chambers were filled with medium supplemented with 20% fetal bovine serum (FBS) as a chemoattractant. Following 16–24 h of incubation at 37 °C with 5% CO_2_, the invading cells adhering to the lower surface of the inserts were fixed and stained with a three-step staining kit (Thermo Scientific, Massachusetts, USA). Images were captured and cell counts were performed under a light microscope (Olympus, Japan) at 100× magnification.

For the scratch assay, treated cells were seeded in 6-well plates and cultured for 48 h until full confluence was achieved. A linear scratch was created in each well using a sterile 10 µL pipette tip, and detached cells were gently removed by washing with phosphate-buffered saline (PBS). Cells were then incubated in serum-free medium to eliminate the confounding effect of FBS on cell migration. Scratch images were acquired at 0 and 24 h using a light microscope (Olympus, Japan). Scratch closure distances relative to the 0 h time point was quantified and analyzed.

### Immunohistochemistry and antibodies

Breast cancer tissues (N = 20) were collected as previously described (33) and subjected to IHC staining and scoring (34). FFPE tissue sections (4 μm) were stained using the EnVision two-step procedure and an IHC kit (PK10006; Proteintech). The primary antibodies used included rabbit anti-TMSB10 antibody (1:100, PA5-116041; Invitrogen). The process was executed according to the manufacturer’s instructions, including the necessary positive and negative controls. IHC staining for the TMSB10 were evaluated by two independent pathologists as described previously (35). The final score between 0 and 3 is low expression, the final score between 4 and 8 is median expression, and the final score greater than 9 is high expression.

### Statistics

All bioinformatics analyzes were conducted by R Studio (v4.2.2, v4.3.3). The corresponding packages were described in Method sections. The student’s t-test was used for comparisons between two groups. Categorical data were assessed with the chi-square or Fisher’s exact test where appropriate. Pearson correlation analysis was used to test the correlation between two continuous variables. The survival differences of different groups were estimated via the log-rank test. A *p*-value less than 0.05 was considered statistically significant.

## Results

### C1 tumor cluster plays an unfavorable role in tumor microenvironment of breast cancer

**Figure 2:**
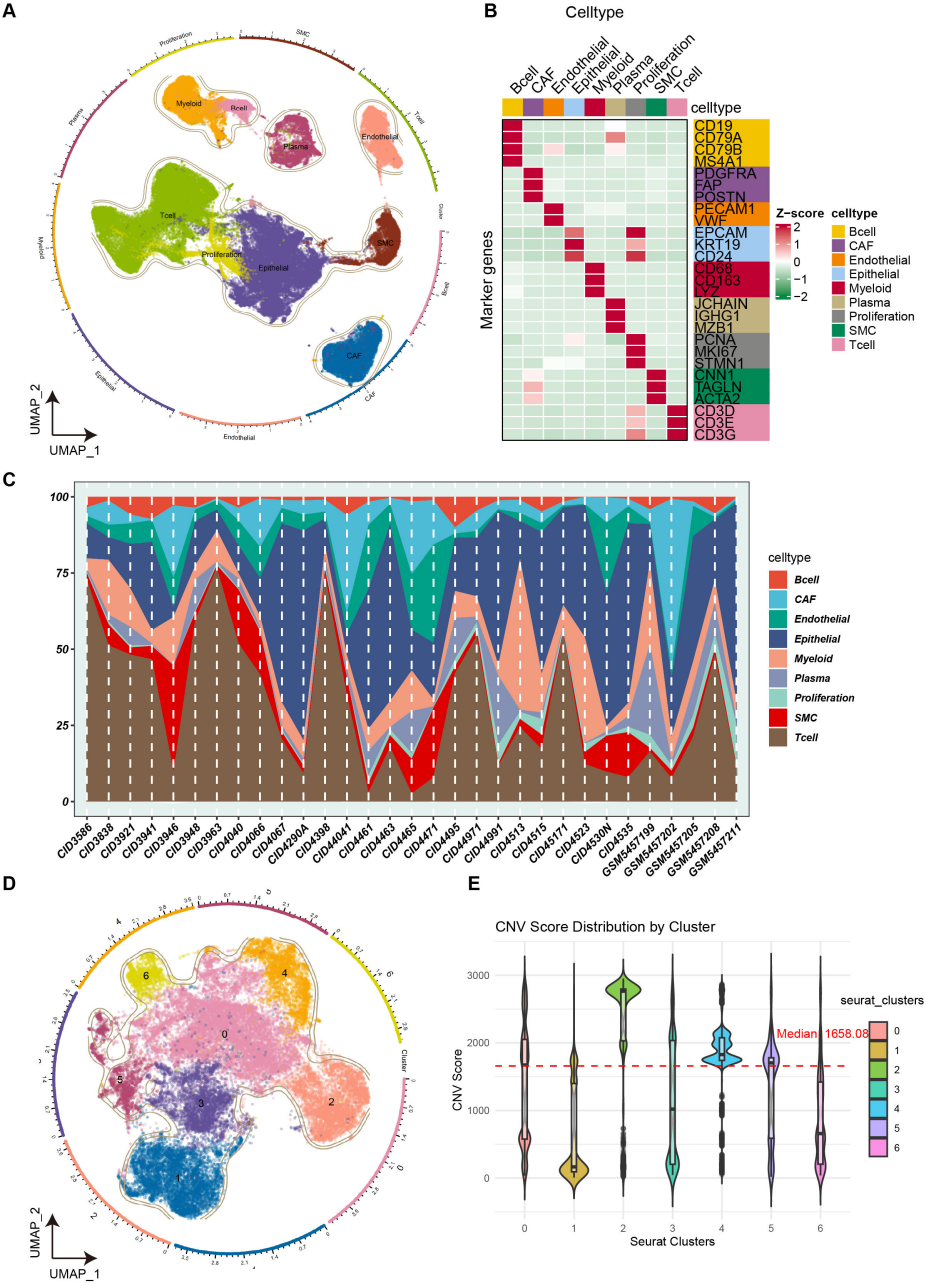
Identification and extraction of malignant cells from breast cancer tissues. **(A)** UMAP scatter plot displays 9 different cell clusters in breast cancer samples. **(B)** The markers used for cell annotation. The color from green to red in the heatmap represents an increase in gene expression **(C)** The infiltration level of distinct cell types in each breast cancer sample. **(D)** UMAP scatter plot displays 7 different epithelial cell clusters in breast cancer samples. **(E)** The violin showing the CNV score of different epithelial clusters. The red dotted line represents the median value of the CNV score.

Collecting a sufficient sample size is a primary condition for analyzing specific subpopulations of tumor cells with an unfavorable prognosis. Thus, we first integrated the scRNA-seq data of 31 primary breast cancers and performed strict quality control as described in the Methods section. The results of quality control were satisfactory and are presented in **Supplementary Figure 1A**. A total of 121,279 high-quality cells were obtained for subsequent analyzes. After normalization and removal of batch effects, the batch effects among samples were significantly reduced (**Supplementary Figure 1B**). The cell clusters were stable when the resolution was set to 0.6 with the assistance of the Clustree package. Ultimately, 32 distinct cell clusters were identified and grouped into 9 main cell types. Nonlinear dimensionality reduction indicated significant differences in expression profiles between cell types (**Figure 2A**). The markers used for cell annotation are presented in **Figure 2B**. The cell abundances of each sample are shown in **Figure 2C**, suggesting significant heterogeneity in the tumor microenvironment of primary breast cancer.

**Figure 3:**
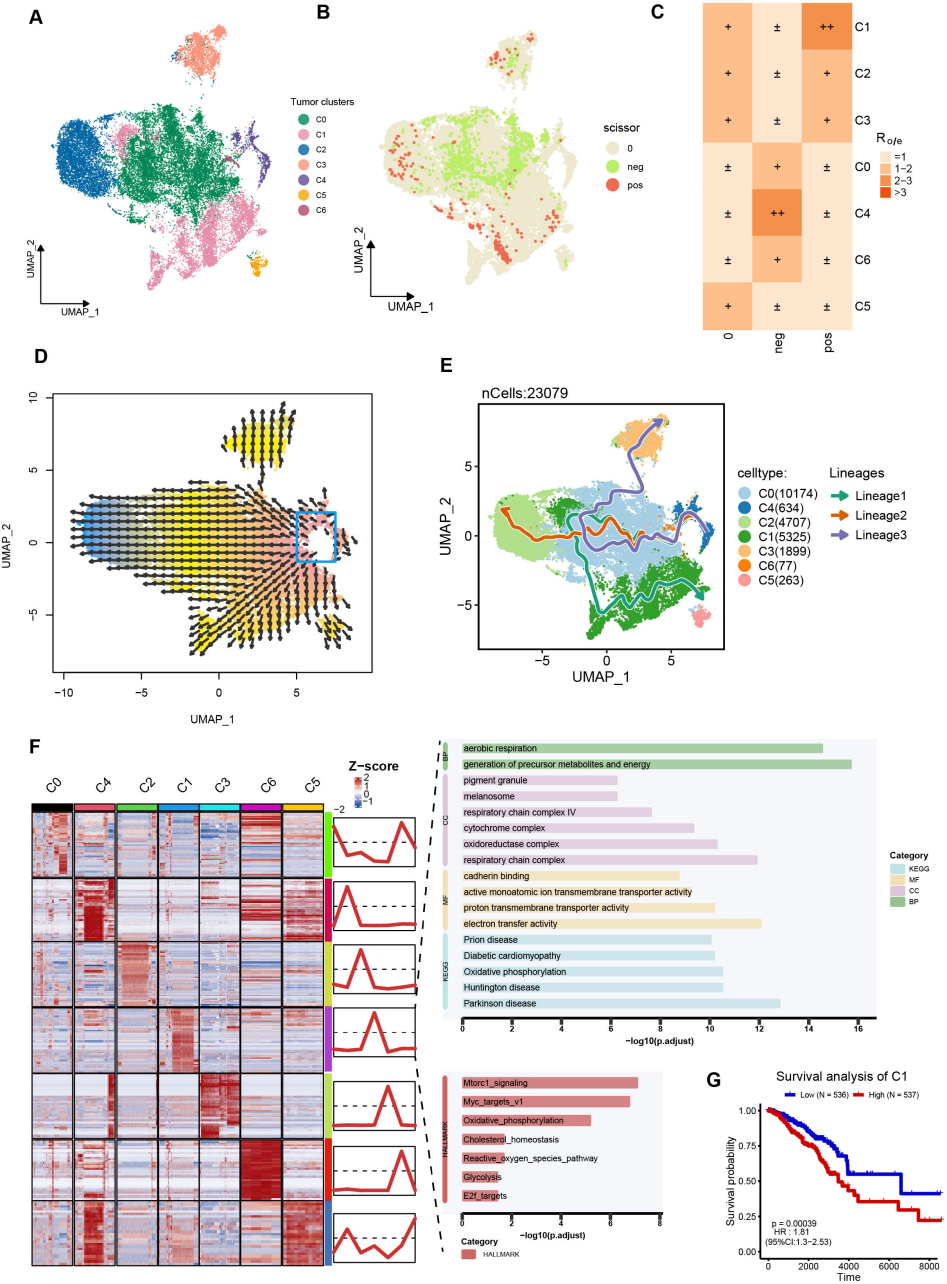
C1 tumor cluster was associated with the unfavorable prognosis of breast cancer patients. **(A)** UMAP scatter plot displays 7 tumor clusters in breast cancer samples. **(B)** UMAP scatter plot presents scissor+ cells and scissor- cells. **(C)** The heatmap showing the tissue preference of scissor+ cells in different tumor clusters. Symbols represent the ratio of observed to expected values. ±, R_obs/exp ≤_ 1; +, 1< R_obs/exp ≤_2; 2< R_obs/exp ≤_3. **(D)** Developmental directions for tumor cells. The arrow indicates the direction of cell development. **(E)** The potential cell trajectory and lineages of tumor cells in tumor microenvironment. **(F)** Heatmap shows the mean of top50 marker genes of clusters. The line graph represents the differential expression of the mean of these marker genes in all clusters. The Z-score represents the normalized numerical value of gene expression levels. **(G)** Kaplan-Meier curves show overall survival (OS) for patients with high or low C1-cell infiltration absolute scores in the TCGA-BRCA cohort.

As tumor cells originate from epithelial cells, we further extracted the epithelial cells and performed the same procedure to cluster these cells. Before grouping, we confirmed that there were no other types of cells mixed in the epithelial cells (**Supplementary Figure 1C**). Seven distinct epithelial cell clusters were identified (**Figure 2D**). To further obtain the tumor cells, the Infercnv algorithm was performed, and clusters 0, 2, 4, and 5 exhibited a high level of CNV score (**Figure 2E**). Therefore, these clusters were considered tumor cells. Subsequently, tumor cells (N = 23,079) were extracted and standardized, and 7 tumor clusters were identified (C1, N = 10174; C2, N = 5325; C3, N = 4707; C4, N = 634; C5, N = 263; C6, N = 77; **Figure 3A**). The Scissor package was used to combine TCGA data (RNA-seq and survival data) with the single-cell RNA matrix to calculate tumor cells associated with prognosis, and scissor+ cells were associated with the unfavorable prognosis of breast cancer (**Figure 3B**). Cell preference analysis revealed that scissor+ cells were enriched in the C1 tumor cluster (**Figure 3C**), suggesting an unfavorable role of the C1 tumor cluster in the breast cancer microenvironment.

We further reconstructed the developmental trajectories of tumor cells to observe potential lineage alterations among tumor clusters. Cell developmental analysis indicated that the C4 cluster was the starting point of tumor cells, suggesting that the C4 cluster might represent early-stage tumor cells (**Figure 3D**). Consistently, another classical algorithm also confirmed these results, with the C1, C2, and C3 clusters located at the end point of the cell trajectory (**Figure 3E**). These results imply that the C1 cluster is associated with the progression of breast cancer. Subsequently, we performed enrichment analyzes to identify the signaling pathways associated with the C1 cluster. As shown in **Figure 3F**, the upregulated genes of the C1 cluster were enriched in respiratory chain complex, electron transfer activity, oxidative phosphorylation, Mtorc1 signaling, MYC target v1, and glycolysis, indicating that the C1 cluster is involved in regulating metabolic reprogramming and malignant cell proliferation. Finally, we utilized deconvolution algorithms to infer the abundance of different tumor clusters in the TCGA cohort and evaluated the prognostic role of the heterogeneity of tumor cluster infiltration levels. Interestingly, the abundance of C1 cluster was the only one associated with poor prognosis in breast cancer (**Figure 3G**, **Supplementary Figures 2A–F**). In summary, these findings suggest that the C1 tumor subgroup is an unfavorable component in the tumor microenvironment of breast cancer, promoting the progression of breast cancer.

### C1 cluster interacts with cancer-associated fibroblasts and endothelial cells to promote the development of TME in breast cancer

**Figure 4:**
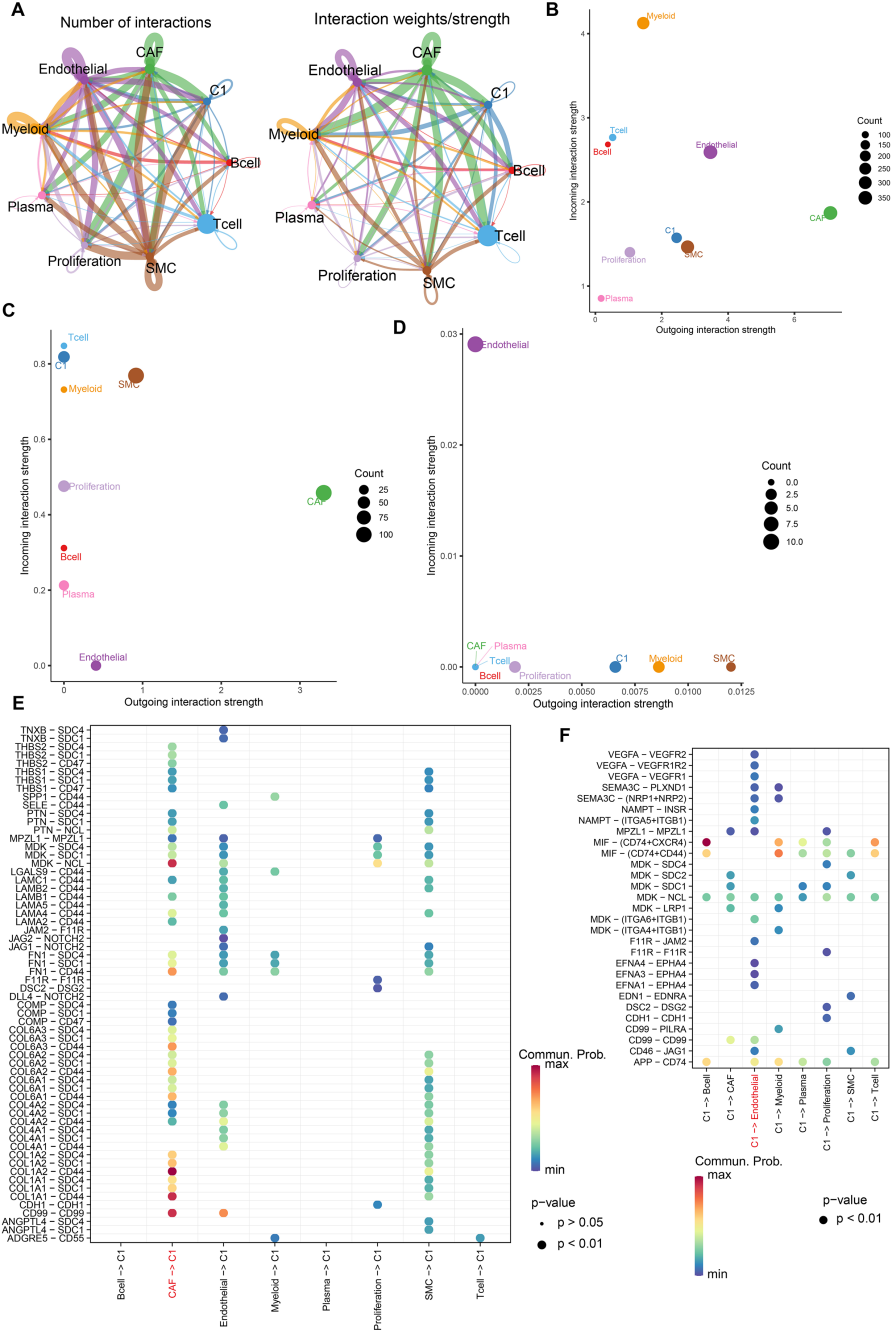
The crosstalk between C1 cluster and other components in tumor microenvironment of breast cancer. **(A)** The number of interactions and interaction weight among cell types in tumor microenvironment. The thickness of the lines represents the strength of the communication. **(B)** The overall interaction strength of the incoming and outcoming signals from distinct cell types. **(C, D)** The interaction strength between collagen **(C)** and VEGF **(D)** signaling pathways in different cell types. **(E)** The plot demonstrating the communication of COLLAGEN signaling pathway between C1 cluster and cancer-associated fibroblasts. A color change from blue to red indicates an increase in communication probability. Dots from small to large represent smaller *p*-values. **(F)** The plot demonstrating the communication of COLLAGEN signaling pathway between C1 cluster and endothelial cells. A color change from blue to red indicates an increase in communication probability. Dots from small to large represent smaller *p*-values.

The above results revealed the poor prognostic effect of the C1 cluster and its intrinsic lineage evolution trajectory. Next, we explored the interactions between the C1 cluster and other components to elucidate its tumor-promoting mechanism in the tumor microenvironment. We analyzed the cell-cell communication between the C1 cluster and other cell types (**Figure 4A**). Globally, cancer-associated fibroblasts (CAFs) and endothelial cells exported the most signals in the breast cancer tumor microenvironment (**Figure 4B**). Therefore, we focused on the interaction between the C1 cluster and these two components. We observed that the C1 cluster received high-strength collagen signals from CAFs (**Figure 4C**) and exported high-strength VEGF signals to endothelial cells (**Figure 4D**). Multiple ligand-receptor pairs, including *COL1A1-CD44, COL1A2-CD44, COL6A1-CD44, COL6A2-CD44*, and *COL6A3-CD44*, exhibited significantly higher communication probability compared to other components (**Figure 4E**), indicating that the C1 cluster can adapt to the tumor microenvironment by sensing mechanotransduction signals from CAFs. Additionally, angiogenic ligand-receptor pairs (*VEGFA-VEGFR2, VEGFA-VEGF1R2, VEGFA-VEGFR1*) specifically exist between the C1 cluster and endothelial cells (**Figure 4F**), suggesting that the C1 cluster promotes angiogenesis in the tumor microenvironment.

**Figure 5:**
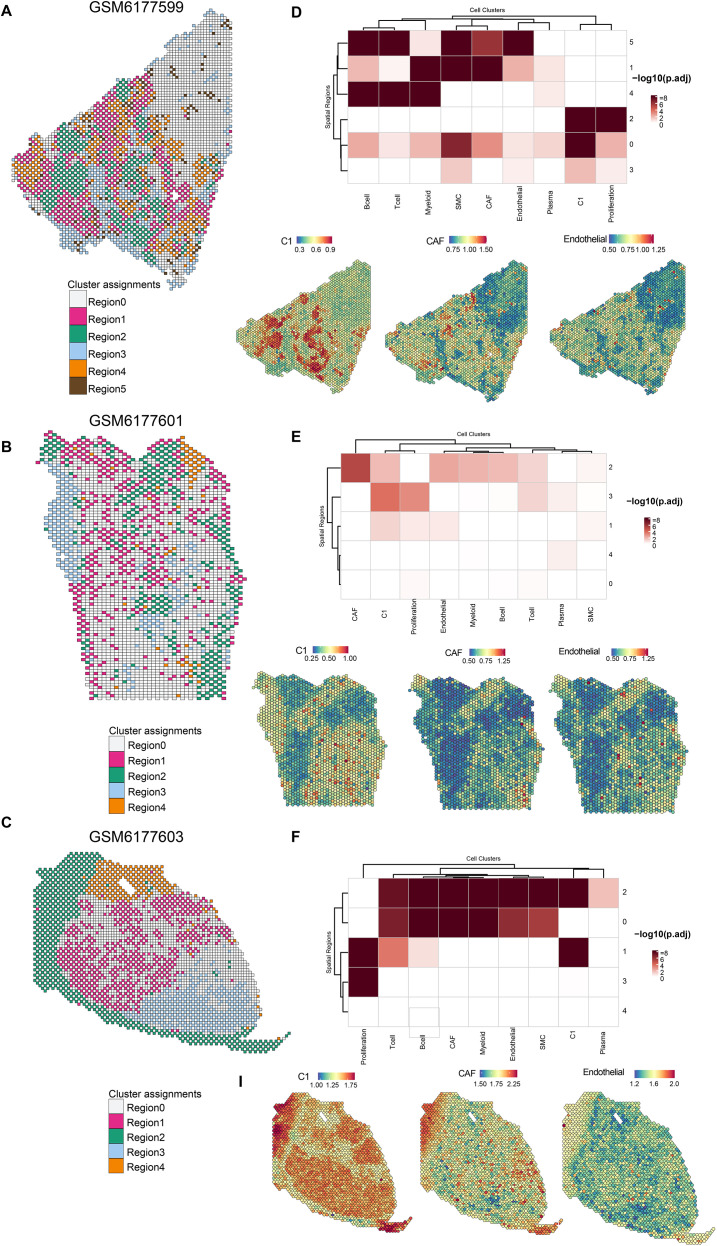
C1 cluster has co-localization relationship with CAFs, cancer-associated fibroblasts and endothelial cells in breast cancer tissues. A-C Regions with different transcriptional profiles were identified in GSM6177599 **(A)**, GSM6177601 **(B)**, and GSM6177603 **(C)**. **(D–F)** MIA, Multimodal intersection analysisand ssGSEA confirming the co-localization relationship between C1 cluster, CAFs and endothelial cells in GSM6177599 **(D)**, GSM6177601 **(E)**, and GSM6177603 **(F)**.

To support our conclusions, we further explored the spatial proximity of these cell subsets in breast cancer. The spatial transcriptomes of breast cancer were standardized, and distinct regions were determined based on the appropriate resolution. As shown in **Figures 5A–C**, the spatial locations in breast cancer tissues were divided into regions with different niches. Subsequently, MIA was utilized to calculate the enrichment score of the C1 cluster and other components using the hypergeometric test. Consistent with the results of cell interaction, we observed that the C1 cluster was significantly highly enriched with CAFs and endothelial cells in specific niches in different breast cancer spatial transcriptome samples (**Figures 5D–F**). Collectively, these findings reveal the potential mechanism of the C1 cluster promoting breast cancer progression from the perspective of the tumor microenvironment.

### Tumor size and genetic mutations associated with the infiltration heterogeneity of C1 cluster

We have confirmed the role of the C1 cluster in breast cancer, and we next explored the factors influencing the infiltration level of the C1 cluster. After excluding samples lacking complete clinical characteristics, a total of 818 samples were included. As shown in **Supplementary Table 5**, age (*p* = 0.764), sex (*p* = 1), race (*p* = 0.603), N stage (*p* = 0.726), M stage (*p* = 0.825), and tumor stage (*p* = 0.114) were not significantly associated with the infiltration level of the C1 cluster. Interestingly, T stage (*p* = 0.014) was significantly associated with the abundance of the C1 cluster, suggesting that the C1 cluster influences the focal invasion of breast cancer.

**Figure 6:**
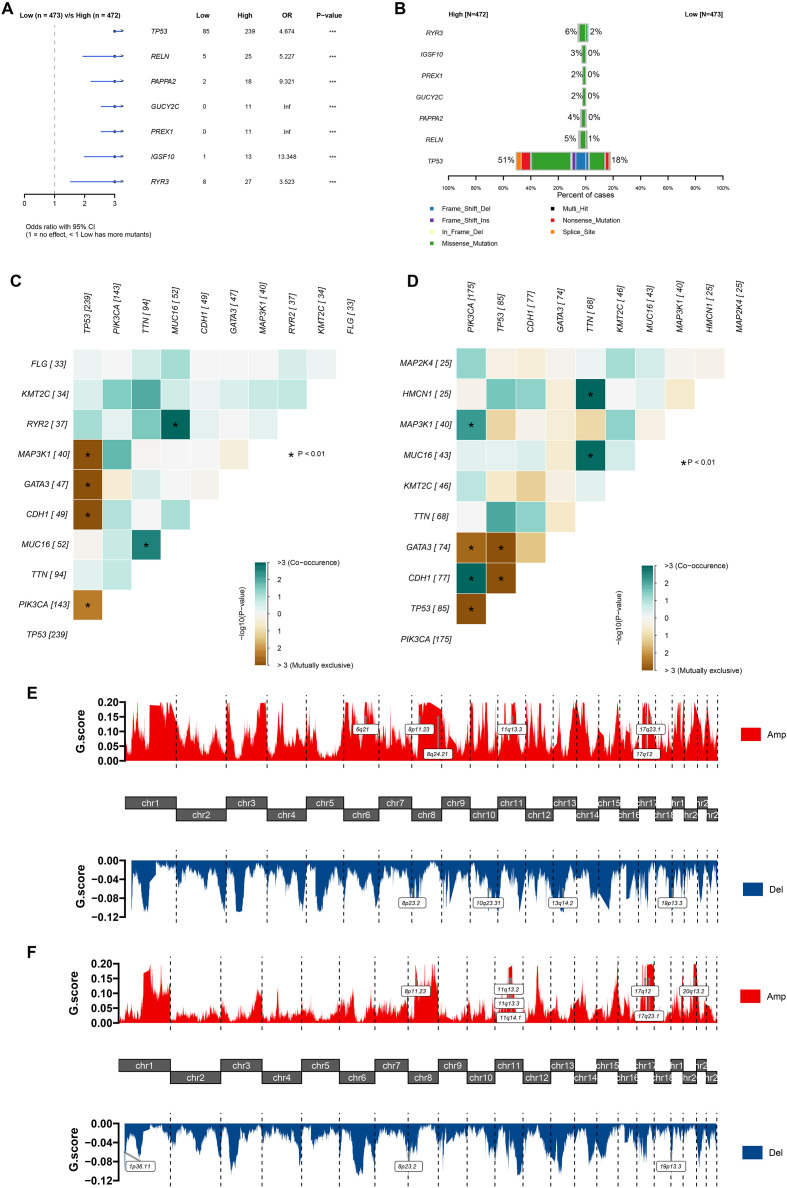
The T stage and focal somatic mutations were associated with the infiltration level of C1 cluster. **(A)** Forest plot illustrating the focal somatic SNV, single nucleotide variant significantly different between patients with high and low C1-cluster infiltrations. OR > 1 indicates a higher SNV frequency in the high C1-cluster infiltration group. OR < 1 indicates a higher SNV frequency in the low infiltration group. **(B)** Bar diagram indicating the frequency of SNVs in the genes highlighted in panel A across the two groups. **(C, D)** Landscape of gene co-mutations in patients with high **(C)** and low **(D)** abundance of C1 cluster. **(E, F)** Visualization of CNAs in patients, stratified by high **(E)** or low **(F)** C1-cluster infiltration levels within chromosomal context. *p<0.01,***p<0.001.

Subsequently, we examined the mutation landscape of the high- and low- groups of the C1 cluster. Focal somatic mutations, including *TP53*, *RELN*, *PAPPA2*, *GUCY2C*, *PREX1*, *IGSF10*, and *RYR3*, were significantly increased in the high C1 cluster group (**Figure 6A**). Notably, these mutations are primarily concentrated in missense mutations (**Figure 6B**). The co-mutation landscape revealed that the high and low groups of the C1 cluster exhibit different co-mutation and co-exclusion characteristics, with the co-mutation phenomenon being more widespread in the low expression group (**Figures 6C, D**). We then characterized the CNV landscape of the distinct groups. Amplifications (8p11.23, 11q13.3, 17q12, 17q23.1) and deletions (8p23.2, 19p13.3) were observed in both high- and low- C1 infiltration groups (**Figures 6E, F**). The amplifications at 6q21 and 8q24.21 were more common in the high infiltration group of the C1 cluster. In summary, these findings highlight the subtle association between somatic mutations, genomic mutations, and the heterogeneity of C1 cluster infiltration, revealing the possible source of the heterogeneity of C1 infiltration.

### Patients with high infiltration level of C1 cluster might benefit from immune checkpoints treatment

**Figure 7:**
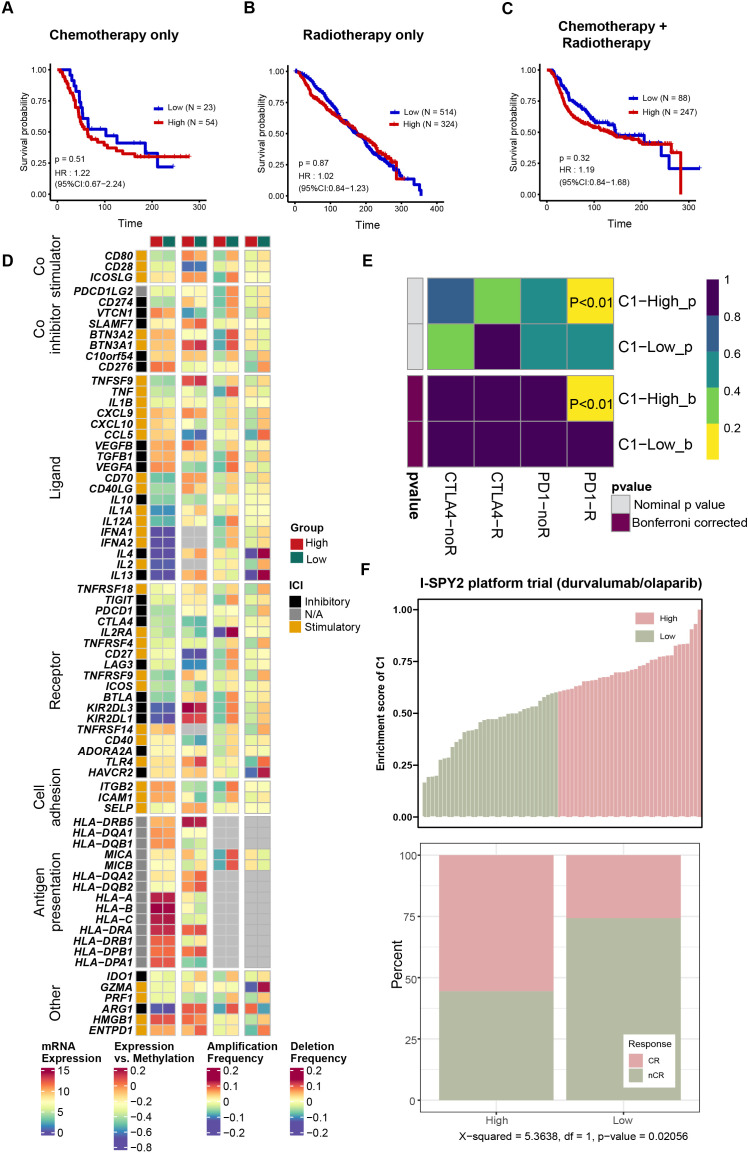
The infiltration level of C1 cluster could be a predictor for immunotherapy. **(A–C)** The Kaplan-Meier graphs revealing the overall survival rates of patients who received chemotherapy alone **(A)**, radiotherapy alone **(B)** and received both radiotherapy and chemotherapy **(C)**. **(D)** Differences in immune regulatory factors between high- and low- C1 groups. **(E)** SubMap analysis predicting differential response to immune checkpoint blockade therapies between the high and low C1 groups. **(F)** The therapeutic responses of the high and low C1 groups to durvalumab combined with olapalib from I-SPY2 platform trial.

The biological functions and invasive heterogeneity of the C1 cluster have been characterized, but its response to different therapies remains unknown. Therefore, we next evaluated the response of the infiltration level of the C1 cluster to various treatments to provide guiding strategies for clinical practice. Samples collected from the METABRIC cohort were regrouped based on distinct treatments, and any sample with incomplete treatment information was excluded. The basis for the grouping was based on the median value of C1 abundance score. For patients who received only chemotherapy (*p* = 0.51, **Figure 7A**), only radiotherapy (*p* = 0.87, **Figure 7B**), or combined chemoradiotherapy (*p* = 0.32, **Figure 7C**), the overall survival rate was not affected by the abundance of the C1 cluster.

Immunotherapy, represented by immune checkpoint inhibitors, has shown great application prospects in the treatment of breast cancer (36). We first mapped the landscape of immunomodulators in different infiltration groups, including expression levels, amplification rates, deletion rates, and correlations between expression levels and methylation (**Figure 7D**), revealing the heterogeneity of the immune microenvironment between the two groups. The submap algorithm confirmed that the expression pattern of patients with high abundance of the C1 cluster was significantly associated with that of patients who responded to PD-1 treatment (*p* < 0.01, **Figure 7E**). Furthermore, breast cancer patients receiving preoperative neoadjuvant duvacizumab/olaparib treatment were classified based on the median enrichment score of the C1 cluster. Consistently, patients with a high enrichment score of the C1 cluster exhibited a higher response rate (*p* = 0.020, **Figure 7F**). In summary, patients with high abundance of the C1 cluster may benefit from immunotherapy instead of chemotherapy or radiotherapy.

### The abundance of C1 cluster could be measured by DCE-T1 MRI images

**Figure 8:**
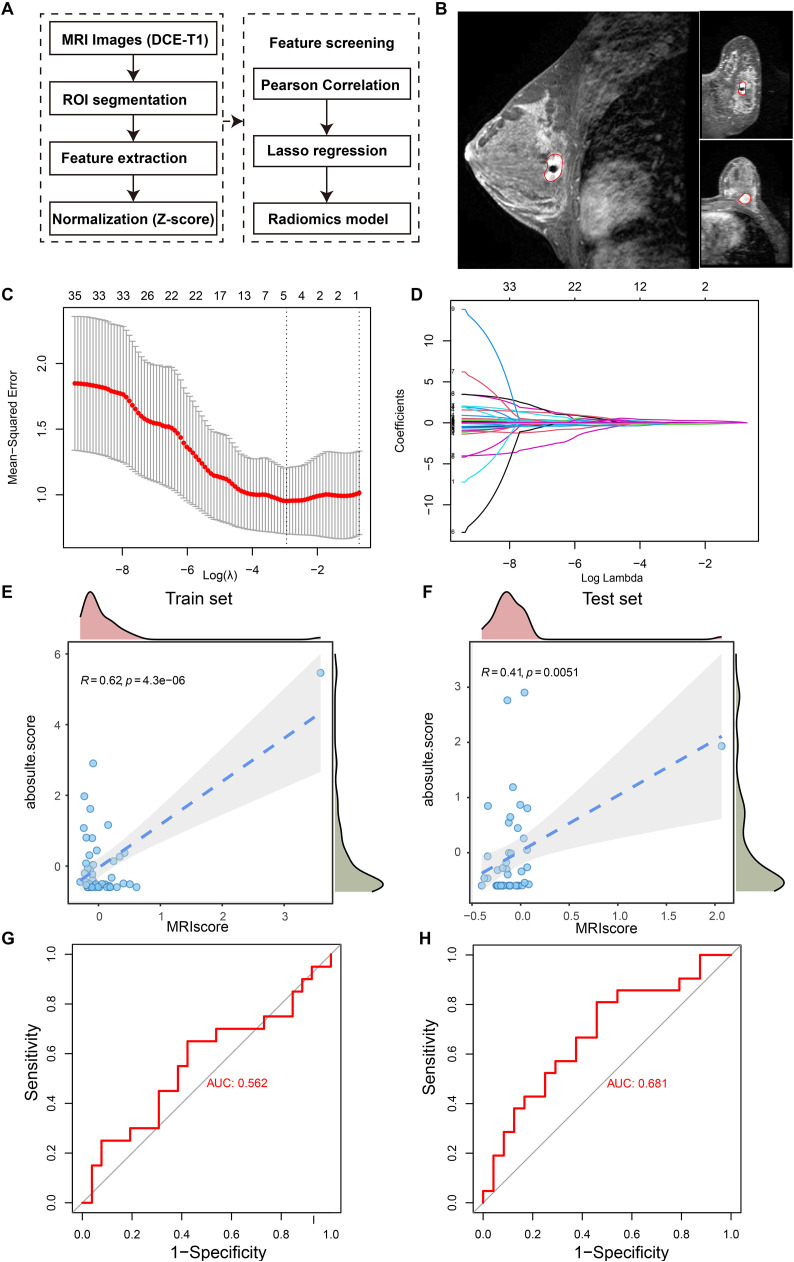
A linear regression model was developed using MRI-based radiomics to estimate the abundance of C1 cluster in breast cancer tissue. **(A)** Schematic diagram illustrating the radiomics analysis process. **(B)** The example shows the segmentation of the tumor area on DCE-T1 MRI image. **(C)** Parameter tuning plot in LASSO, least absolute shrinkage and selection operator regression. **(D)** Distribution of coefficients for variables in the LASSO regression. Each curve represents a radiomics feature filtered by Pearson’s correlation. **(E, F)** Pearson’s correlations between z-score normalized C1 cluster abundance measured by the transcriptome and the fitted value by the linear regression radiomics model in the training set **(E)** and test set **(F)**. **(G, H)** The ROC, receiver operating characteristic curves indicating the model’s ability to discriminate the abundance of C1 cluster in the training set **(G)** and test set **(H)**.

The abundance of the C1 cluster was confirmed as an important indicator for prognosis and immunotherapy. It is promising to further develop a practical tool for evaluating its clinical translation. We hypothesized that the abundance of the C1 cluster could be observed via MRI-based radiomics. A total of 91 TCGA-BRCA samples with DCE-MRI data and RNA-seq were included. The overall design of the radiomic model construction is shown in **Figure 8A**. The regions of interest (ROI) were manually segmented by two breast oncologists using 3D slicer (**Figure 8B**). Over 800 features of MRI images were obtained. Subsequently, the samples were randomly divided into the training set (N = 46) and the validation set (N = 45) in a 1:1 ratio. To further obtain high-priority features, Pearson correlation analysis was performed, and 38 candidate features were obtained for subsequent analysis (p < 0.05, **Supplementary Table 6**). Due to the limitation of the sample size, we chose conservative linear regression to construct the model. Finally, five candidate features and their corresponding coefficients were determined (**Figures 8C, D**, **Supplementary Table 7**). Interestingly, the radiomic score was closely associated with the absolute score of the C1 cluster in both the training set (R = 0.62, *p* < 0.001, **Figure 8E**) and the validation set (R = 0.41, *p* < 0.001, **Figure 8F**). Receiver operating characteristic (ROC) curve analysis showed that the area under the curve of the radiomics score for differentiating the infiltration level of the C1 cluster in the training set (**Figure 8G**) and the validation set (**Figure 8H**) was 0.563 and 0.681, respectively. The results indicate that the abundance of the C1 cluster in the tumor microenvironment could be measured by a radiomic model based on MRI images, providing a non-invasive strategy for estimating the abundance of the C1 cluster.

### Construction of a robust prognostic model for risk stratification based on C1 cluster

**Figure 9:**
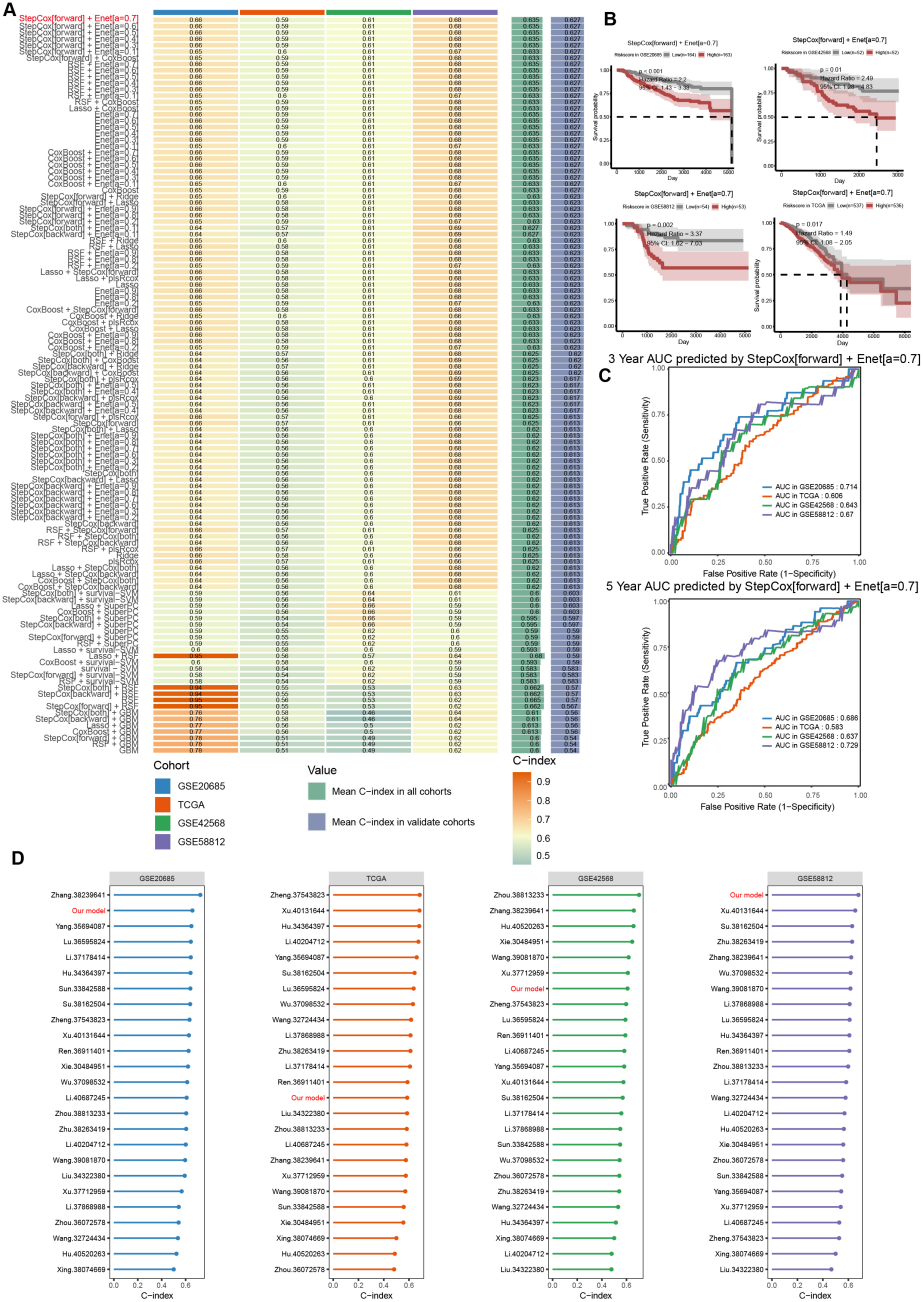
Construction of prognostic model based on the biomarkers of C1 cluster using machine learning algorithms. **(A)** Construction of the prognostic model based on C1 makers using machine learning techniques, optimizing the model to predict patient outcomes. **(B)** Survival analysis comparing high and low C1 risk groups in distinct datasets. **(C)** ROC curve analysis of the prognostic model in 3- and 5- years survival. **(D)** Comparison of the C-index of the prognostic model with publicly prognostic models.

To further explore the clinical translational value of the C1 cluster, we developed a prognostic model for breast cancer risk stratification. The top 50 C1 cluster markers were identified through differential expression analysis (**Supplementary Table 8**). The GSE20685 cohort (N = 327) was used as the training set, while the TCGA-BRCA cohort (N = 1073), GSE42568 cohort (N = 104), and GSE58812 cohort (N = 107) were used as validation sets. Over 100 machine learning algorithms were used to construct robust cross-dataset prognostic models. Univariate Cox regression was conducted, and 11 genes (*TMSB10, PGK1, ATG5, CD24, S100A9, PPIA, S100A8, PERP, P4HB, KLF6, CFL1*) were selected for model construction. The concordance index (C-index) was the highest in the validation set when combined with the stepCox(forward) and elastic net regression (α = 0.7) algorithms (**Figure 9A**). We also observed that the risk score could significantly distinguish the prognosis of breast cancer patients (**Figure 9B**). Additionally, the model we constructed showed potential for predicting the 3- and 5-year overall survival rates of breast cancer patients (**Figure 9C**; **Supplementary Figures 3A, B**). Subsequently, we collected publicly available prognostic models of breast cancer and compared our model with them. As illustrated in **Figure 9D**, the predictive ability of our model demonstrated stronger consistency in the same training and validation cohorts compared to the publicly published models. Overall, this prognostic model based on the C1 cluster demonstrates robustness and potential for clinical application in breast cancer risk stratification.

### TMSB10 as the core gene of C1 cluster, inhibited cell proliferation, invasion, and migration

**Figure 10:**
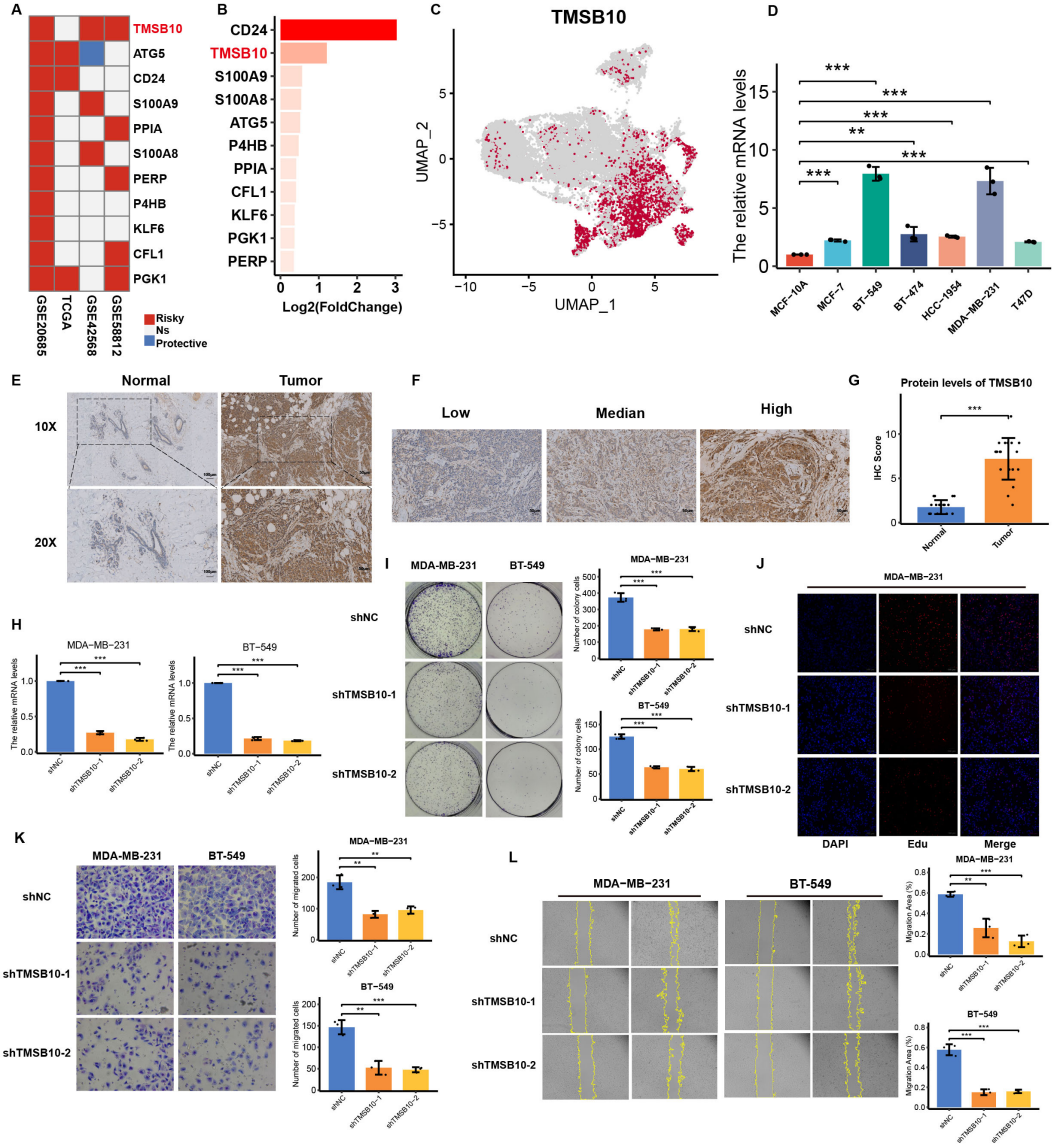
Experimental validation of TMSB10 expression and function in breast cancer. **(A)** The hazard ratio of candidate genes of C1 cluster in distinct datasets. **(B)** The fold change in the expression level of candidate genes in the C1 cluster compared to other tumor clusters. **(C)** The expression level of TMSB10 in all tumor cells. **(D)** The mRNA level of TMSB10 in breast cancer cell lines detected by RT-qPCR. **(E)** The protein expression level of TMSB10 in 20 paired normal and breast cancer were detected by IHC. **(F)** The standard score of IHC staining. **(G)** The IHC scores of TMSB10 by IHC staining. **(H)** RT-qPCR analysis of TMSB10 expression levels in MDA-MB-231- shTMSB10 cells and BT-549- shTMSB10 cells compared with shNC cells, respectively. **(I-L)** The cell proliferation assays and invasion assays were conducted, including, colony formation assay **(I)**, EdU assay **(J)**, transwell assay **(K)**, scratch assay **(L)**. All cell experiments were biologically repeated three times (*n* = 3). Error bars represent SD. **p < 0.01; ***p < 0.001.

The above findings highlighted the critical role and clinical translational value of the C1 cluster. Therefore, identifying the biomarkers of the C1 cluster is of crucial importance for preclinical research. We observed that the adverse prognostic effects of *TMSB10* and *PGK1* in the candidate genes were more widespread across multiple cohorts (**Figure 10A**). By combining the overexpressed genes of the C1 cluster, we identified *TMSB10* as a potential core gene of the C1 cluster (**Figure 10B**), which was also validated by scRNA-seq (**Figure 10C**; **Supplementary Figure 4A**). The prognostic value and immunosuppressive role of TMSB10 were validated by Kaplan-Meier plotter and GEPIA2 databases (**Supplementary Figures 4B, C**). Of note, enrichment analysis indicates that the biological functions of *TMSB10* and the C1 cluster are highly consistent (**Supplementary Figures 4D, E**).

The mRNA level of TMSB10 was significantly upregulated in breast cancer cell lines compared to the normal cell line (MCF-10A, **Figure 10D**). Consistently, the protein level of *TMSB10* was observed to be highly expressed in breast cancer tissues (**Figures 10E–G**). To investigate the role of TMSB10 in breast cancer progression, stable *TMSB10*-knockdown MDA-MB-231 and BT-549 cell lines were established, with the knockdown efficiency validated via RT-qPCR (**Figure 10H**). Compared with the control group, *TMSB10*-knockdown cells exhibited impaired colony-forming capacity (**Figure 10I**), and a lower proportion of EdU-positive cells (**Figure 10J**; **Supplementary Figures 5A–C**), demonstrating that *TMSB10* promotes breast cancer cell proliferation. Consistently, functional assays including the transwell invasion assay (**Figure 10K**) and the cell scratch assay (**Figure 10L**) revealed that *TMSB10* depletion led to attenuated invasive potential in these breast cancer cells. Collectively, these findings indicate that *TMSB10* promotes breast cancer cell proliferation, invasion, and migration *in vitro*.

## Discussion

Our research provides a comprehensive multi-omics study, depicting a high-risk tumor cell cluster C1 and establishing its key molecular effector *TMSB10* as a core driver of breast cancer progression. By integrating single-cell transcriptomics, spatial transcriptomics, bulk RNA data, genomic data, and *in vitro* experiments, we demonstrate that the C1 cluster acts as a critical driver for tumor progression, influencing clinical outcomes through intrinsic metabolic reprogramming and systematic remodeling of the tumor microenvironment. Its clinical relevance is significantly associated with poor prognosis, specific somatic mutations and a high response to immunotherapy. To establish the connection between biological discoveries and clinical applications, we further developed non-invasive radiology and robust prognostic models, and provided direct experimental validation for the tumor-promoting function of *TMSB10*, offering a research framework for breast cancer from cellular characterization to therapeutic insights.

The recent application of single-cell and spatial transcriptomics has profoundly expanded our understanding of the cellular ecosystem in multiple cancers (37, 38), successfully revealing previously obscured landscapes of cellular crosstalk (39), clonal evolution (40), and microenvironmental heterogeneity (41). However, significant challenges remain in translating these complex biological features into practical clinical practice, often due to fragmented analytical approaches that cannot effectively connect distinct omics data. Our study was designed to address this specific translational gap. We leveraged a series of computational algorithms from a deliberately clinical perspective. Starting with the identification of malignant epithelial cells based on DNA copy number variation patterns, we employed the Scissor algorithm (42) to directly integrate single-cell transcriptional profiles with bulk RNA-seq data and patient survival information. This analysis revealed a significant enrichment of ‘scissors+’ cells with poor prognosis in the C1 cluster, identifying them as a key cell population for tumor invasiveness. Therefore, through transcriptome deconvolution estimation, the abundance of cluster C1 is an independent prognostic factor associated with poor prognosis in patients. Biologically, the C1 cluster is characterized by the coordinated activation of metabolic processes and the cell cycle, including oxidative phosphorylation (43), mTORC1 signaling (44), MYC targets (45), and glycolysis (18), indicating its profound involvement in metabolic reprogramming and thereby exacerbating its malignant phenotype.

Although previous studies have successfully identified different and specific subtype tumor subpopulations. For example, Karaayvaz et al. (46) identified a tumor subgroup with high expression of SPTLC1 related to glycosphingolipid metabolism by integrating TNBC samples from 6 patients, which could predict the poor prognosis of triple-negative breast cancer. A recent study (47) also identified a specific tumor subpopulation overexpressing NENF that promotes the metastasis of TNBC. Focusing on the tumor subgroups of a single subtype of breast cancer can enhance the efficiency of precision treatment, but it also leads to a loss of a comprehensive understanding of the overall breast cancer tumor cells. Our research benefited from a larger and more comprehensive cohort, which identified the C1 cluster as a universal and aggressive subgroup, indicating a more universal role in disease progression in the broader breast cancer spectrum. In addition to its independent prognostic value, the cell development and trajectory analyzes indicate that C1 cluster may represent a late evolutionary state and may originate from other tumor subgroups. Furthermore, cell-cell communication and spatial co-localization analyzes indicated that the C1 cluster actively participates in the stromal environment by receiving pro-tumor biomechanical signals from cancer-associated fibroblasts and stimulating angiogenesis by transmitting VEGF signals to endothelial cells. These findings collectively explain a dual mechanism of action, namely that the C1 cluster drives tumor growth through intracellular pathways and systemic microenvironment remodeling.

To further explore the clinical translational significance of the C1 cluster, we systematically explored the factors related to its infiltration heterogeneity and its impact on therapeutic effects. Interestingly, we found that the infiltration level of the C1 cluster was correlated with T stage, but not with age, race, tumor stage, M stage, or N stage, which indicates that the C1 cluster was associated with local tumor invasion. At the genomic level, somatic mutations of key genes such as *TP53, RELN, PAPPA2, GUCY2C, PREX1, IGSF10* and *RYR3* are significantly enriched in tumors with high C1 infiltration, providing a potential genetic explanation for the observed inter-tumor heterogeneity. From a therapeutic perspective, although the abundance of C1 cluster cannot predict survival differences in patients receiving conventional radiotherapy or chemotherapy, it shows a strong and consistent association with the response to immune checkpoint blockade. Specifically, the global transcriptional profile of high-C1 patients is significantly similar to that of patients known to respond to PD-1 inhibition. This finding was further confirmed in a clinical cohort of breast cancer patients receiving neoadjuvant durvalumab and olaparib, where a higher C1 enrichment score was significantly associated with a higher pathological complete response rate. These convergent pieces of evidence strongly suggest that C1 abundance is a promising predictive biomarker guiding immunotherapy decisions.

MRI- and CT-based radiomics have emerged as an effective tool for individualized diagnosis and response prediction, which was reported in several tumors including cervical cancer (48), lung cancer (49), and breast cancer (50). We attempted to establish the connection between the microscopic effects and imaging features of cluster C1. Acknowledging the current scarcity of samples with paired imaging and transcriptomic data, our development of a radiomic signature from DCE-MRI represents an exploratory but significant step. This signature maintained a significant correlation with C1 abundance in both training and validation sets, supporting the fundamental feasibility of estimating this key biological feature from routine medical images. The integrated application of machine learning is currently an effective tool for the development of biomarkers and prognostic models. For instance, Zhang et al. (51) identified that SLC2A1 was the promising biomarker for lung adenocarcinoma by integrating more than 100 machine learning algorithms. We also used a variety of machine learning algorithms and their combination based on signature genes of C1 cluster to construct a robust prognostic model across high-throughput sequencing and microarray to assess the overall survival of breast cancer patients. Compared with other publicly available models, this model demonstrates robust consistency across multiple datasets. This indicates that the model developed based on the C1 characteristic gene is a potential prognostic stratification tool for breast cancer patients, especially in predicting the 3-year and 5-year survival rates of patients. And it represents a clinically applicable risk stratification tool that is directly rooted in clearly defined and biologically aggressive tumor subpopulation.

Our focus on the C1 cluster naturally led us to identify *TMSB10* as a central and highly expressed gene within it. Previous studies have suggested a role for *TMSB10* in breast cancer progression. For instance, Zhang et al. (52) found that *TMSB10* promotes the progression of breast cancer through *AKT/FOXO* signaling pathway and can be used as a serum biomarker for breast cancer patients. Bouchal et al. (53) reported that *TMSB10* is associated with the aggressive tumor phenotype of high-grade breast cancer at both protein and transcriptional levels. Our results showed that high expression level of *TMSB10* was associated with the cell cycle, oxidative phosphorylation and EMT, which is consistent with the function of the C1 cluster we previously discovered. We demonstrated that knockdown of *TMSB10* can effectively inhibit the proliferation, migration and invasion of breast cancer cells *in vitro*. These results clearly establish that *TMSB10* is not only a passive marker but also an active functional mediator of the C1 cluster invasive phenotype, thereby highlighting its great potential as a therapeutic target. However, the molecular mechanism of *TMSB10* regulation of tumor metabolism, progression and clinical transformation application are still needed to be further explored in future research.

Although this study based on plentiful multi-scale data provides a broader and comprehensive perspective on breast cancer, we still need to clarify some limitations of the study. Only three spatial transcriptome samples were included in this study. With the continuous reduction of sequencing costs and the development of sequencing technology, more spatial transcriptome samples with higher spatial resolution need to be included in the future, and multiplex immunofluorescence staining techniques should be applied to confirm the spatial co-localization relationship between cells. In addition, we used CIBERSORTX to infer the infiltration level of cells. Although CIBERSORTX has been reported as the current anti-deconvolution algorithm that is closest to real-world data compared to other algorithms (54), the deconvolution algorithm cannot accurately quantify the level of cell infiltration completely and will introduce some inevitable bias. For the diagnostic model constructed based on MRI, we admit that this is an exploratory study and cannot guarantee its accuracy in clinical applications. This is just the first step of our study, and we plan to collect more independent external imaging cohorts to validate our model in subsequent research. Similarly, the candidate genes of the prognostic model need to be further streamlined to expand its potential for clinical application. More importantly, this study lacked sufficient experiments to explore the molecular mechanism of *TMSB10*, although this was not the main purpose of this study. A variety of *in vitro* and *in vivo* experiments should be conducted for investigating its biological functions in subsequent research. Under the background of medical technology advancement and open academic environment, we believe that in addition to common RNA sequencing, rare data including proteomics, pathology, immunology libraries, etc., will be open access in the future, which will play an important role in breast cancer heterogeneity analysis.

## Data Availability

The original contributions presented in the study are included in the article/**Supplementary Material**. Further inquiries can be directed to the corresponding authors.
