# Supplementary material for: Integrative multi-omics and radiomics reveal a TMSB10-driven cell state for non-invasive assessment and precision stratification in breast cancer
Source: Front Immunol. 2026 Apr 20;17:1794329. doi: 10.3389/fimmu.2026.1794329 (PMC13136269; doi:10.3389/fimmu.2026.1794329)
Supplement: Supplementary file 1 [file Table1.docx]

**Supplementary Material**

**Integrative Multi-omics and Radiomics Reveal a TMSB10-Driven Cell State for Non-invasive Assessment and Precision Stratification in Breast Cancer**

Gui-Xin Wang^1,2,3,4#^, Jun-Ming Cao^1,2,3,4#^, Cheng-Lu Lu^5#^, Yun-Lin Wang^6#^, Zi-Yi Chen^7^, Chang-Qing Yang^8^, Shuo Wang^1,2,3,4,8^, Zhang-Yin Guo^1,2,3,4^, Yue Yu^1,2,3,4*^, Shan Cheng^1,2,3,4*^, Xin Wang^1,2,3,4*^

**Supplementary Figure**

**Figure S1**

**
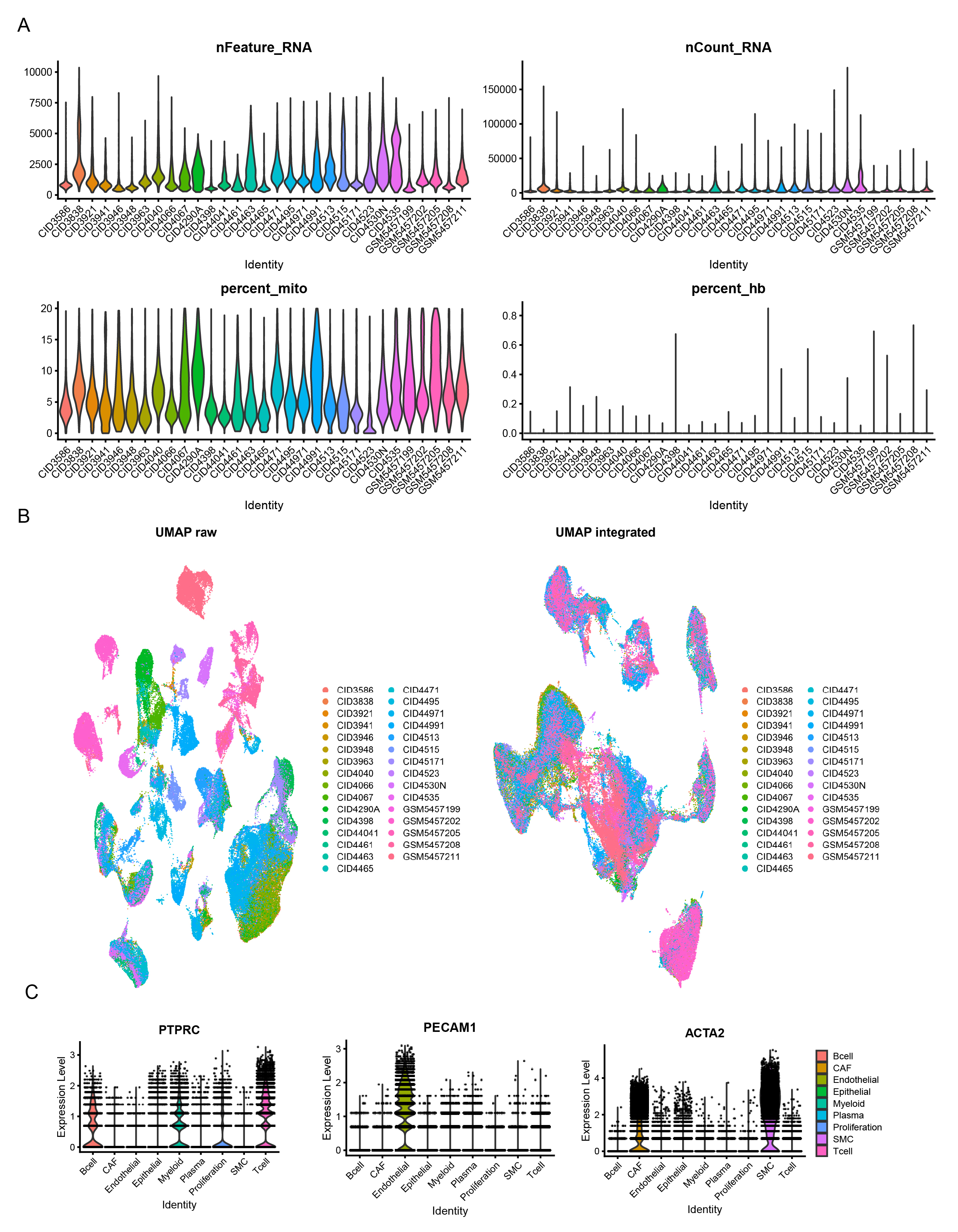
**

**Figure S1 The quality control of single-cell RNA sequencing (scRNA-seq) of breast cancer samples. A** The violin plots showing the strict standard criteria of scRNA-seq in the number of genes detected per cell, total number of molecules detected per cell, proportion of mitochondrial genes, and proportion of hemoglobin genes. **B** UMAP scatter plots showed the batch effect was removed after using harmony algorithm. **C** The expression level of cell specific markers for immune cells (PTPRC), endothelial cells (PECAM1) and stromal cells (ACTA2).

**Figure S2**


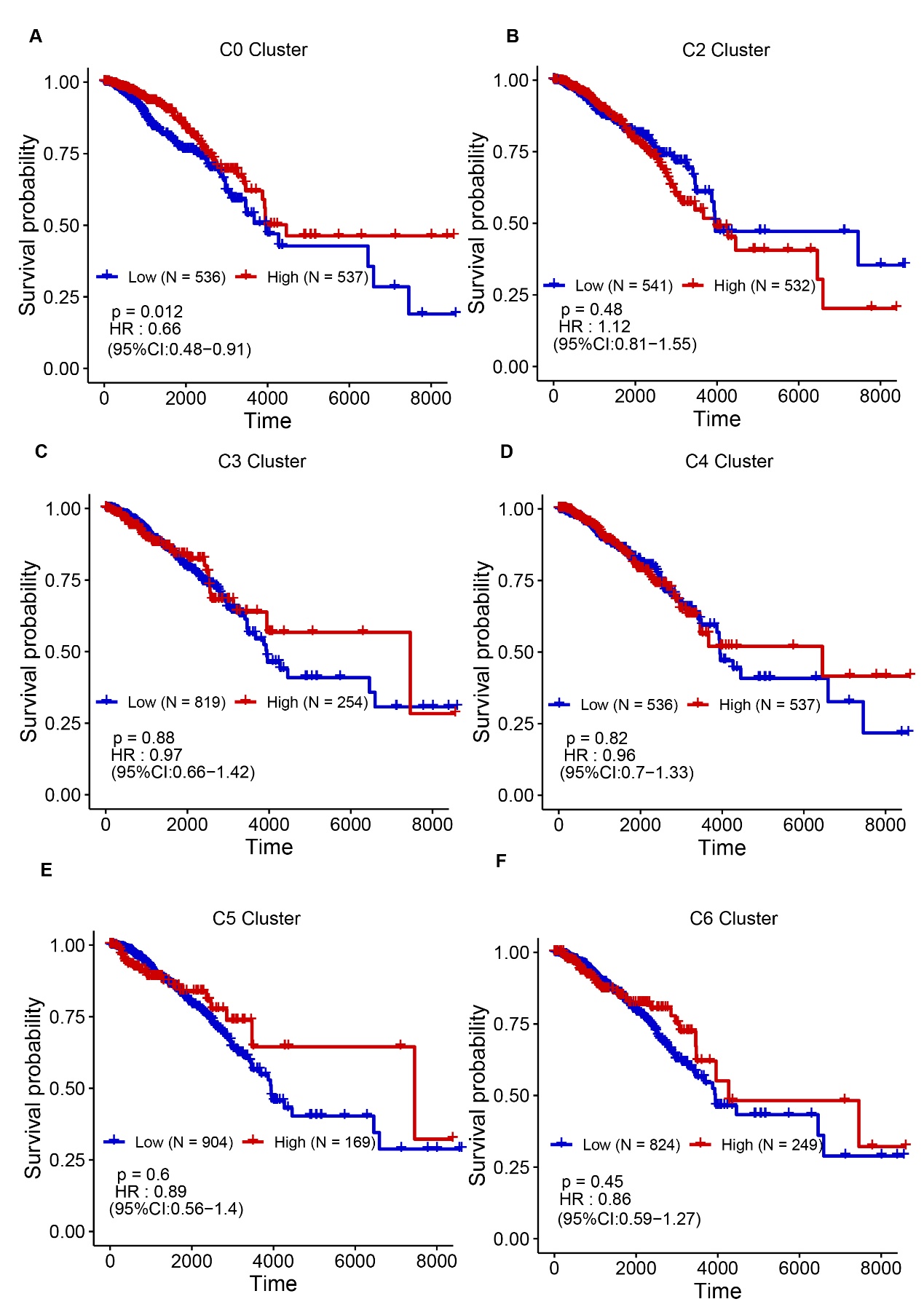


**Figure S2 The prognostic role of tumor clusters based on their infiltration level. A-F** Kaplan-Meier curves show overall survival (OS) for patients with high- and low- C0-cell infiltration absolute scores **(A)**, C2-cell infiltration absolute scores **(B)**, C3-cell infiltration absolute scores **(C)**, C4-cell infiltration absolute scores **(D)**, C5-cell infiltration absolute scores **(E)**, C6-cell infiltration absolute scores **(F)** in the TCGA-BRCA cohort.

**Figure S3**


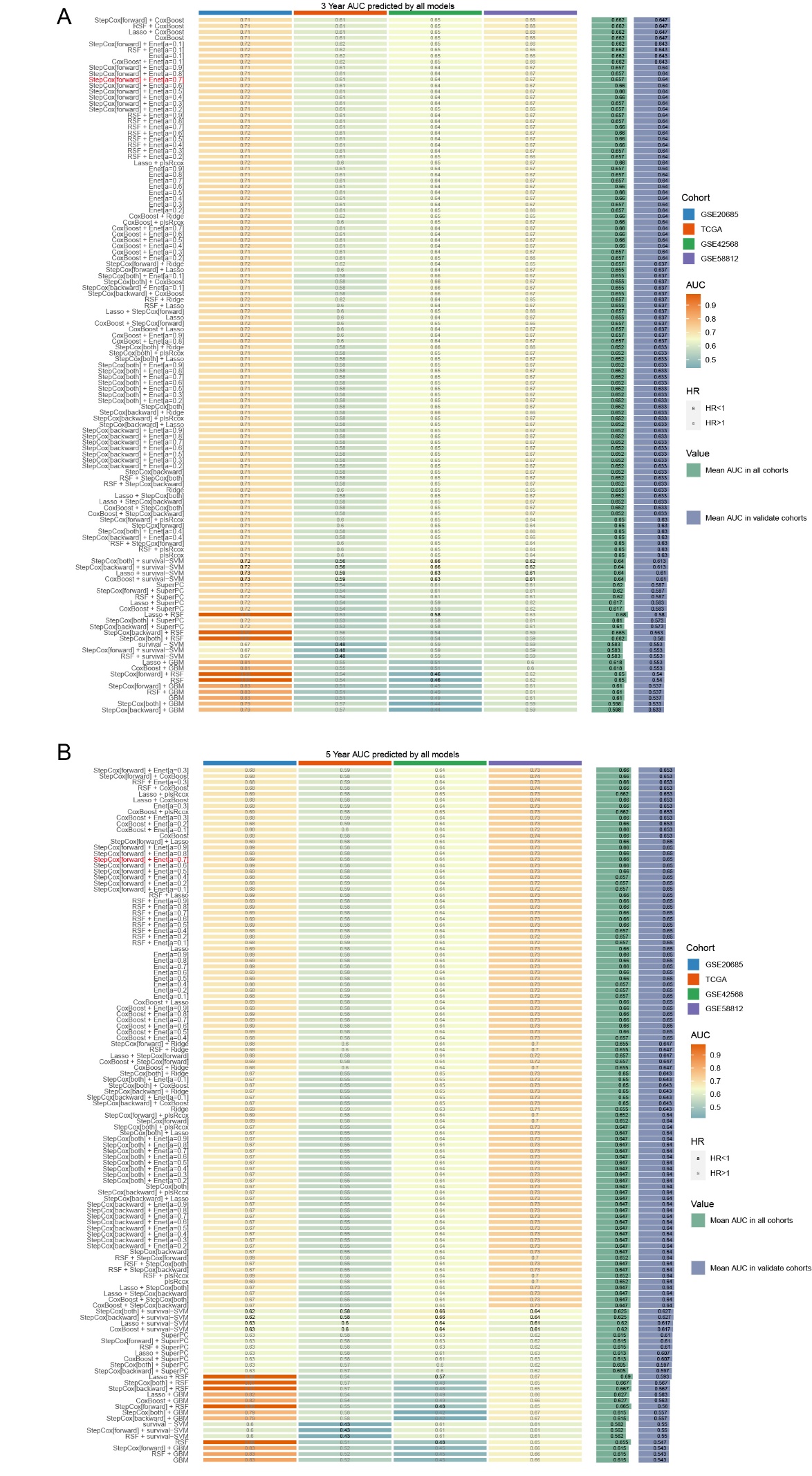


**Figure S3 The discriminatory ability of the model. A-B** The 3 year- (**A**) and 5 year- (**B**) Area Under Curve (AUC) of the prognostic model performed by multiple algorithms in all datasets.

**Figure S4**


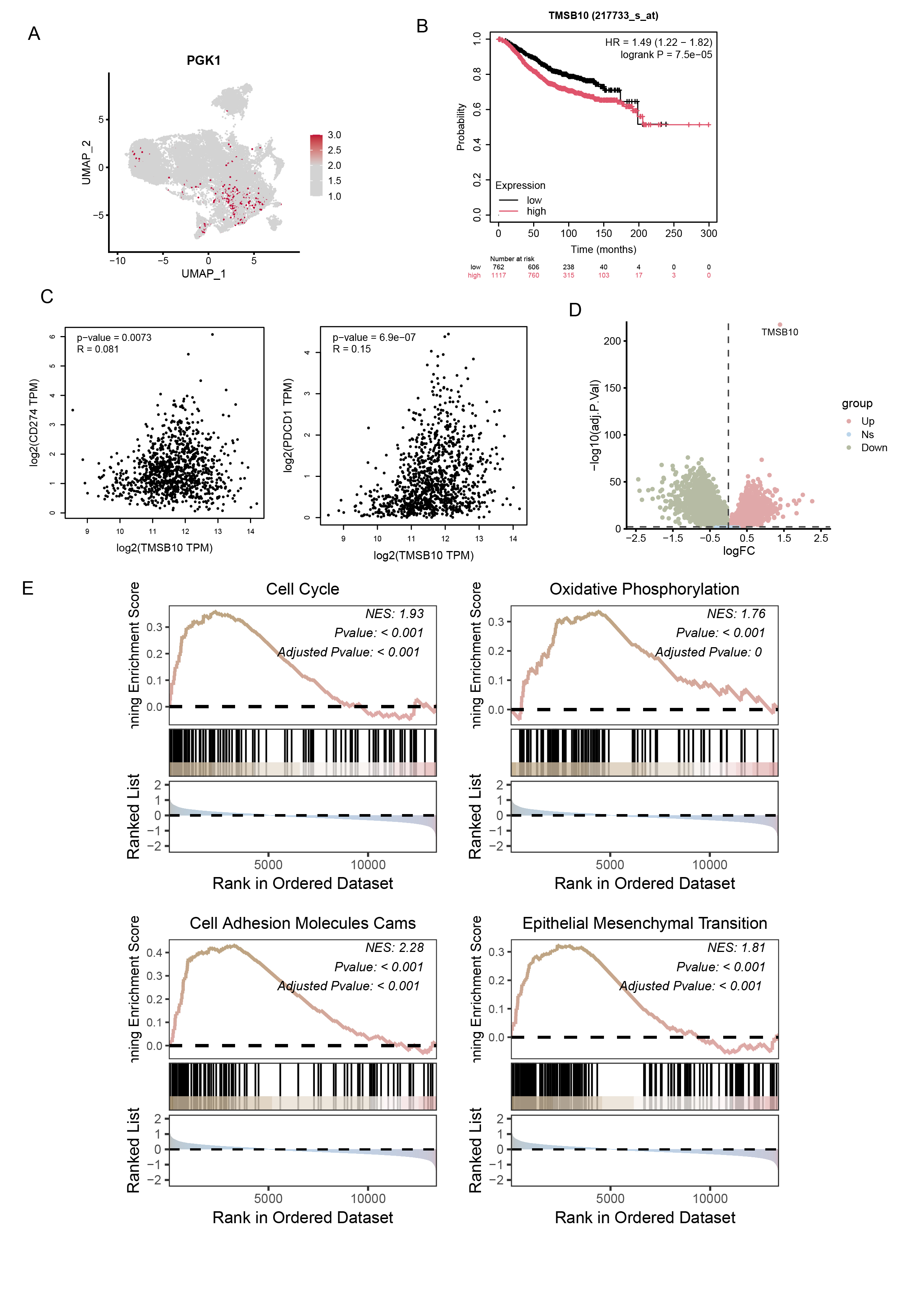


**Figure S4 The potential signaling pathways associated with TMSB10. A** UMAP scatter plots displayed the expression of PGK1 in malignant cells. **B** Survival analysis displayed the unfavored prognosis of TMSB10 in breast cancer in Kaplan-Meier plotter database. **C** Correlations between the expression of TMSB10, and immune checkpoints. **D** Volcano map showing the differentially expressed genes between high- and low- C1 patients inn TGCA-BRCA cohort. **E** GSEA plots showing the enrichment of cell cycle, oxidative phosphorylation, cell adhesion molecules Cams, epithelial mesenchymal transition associated with high expression level of *TMSB10*.

**Figure S5**


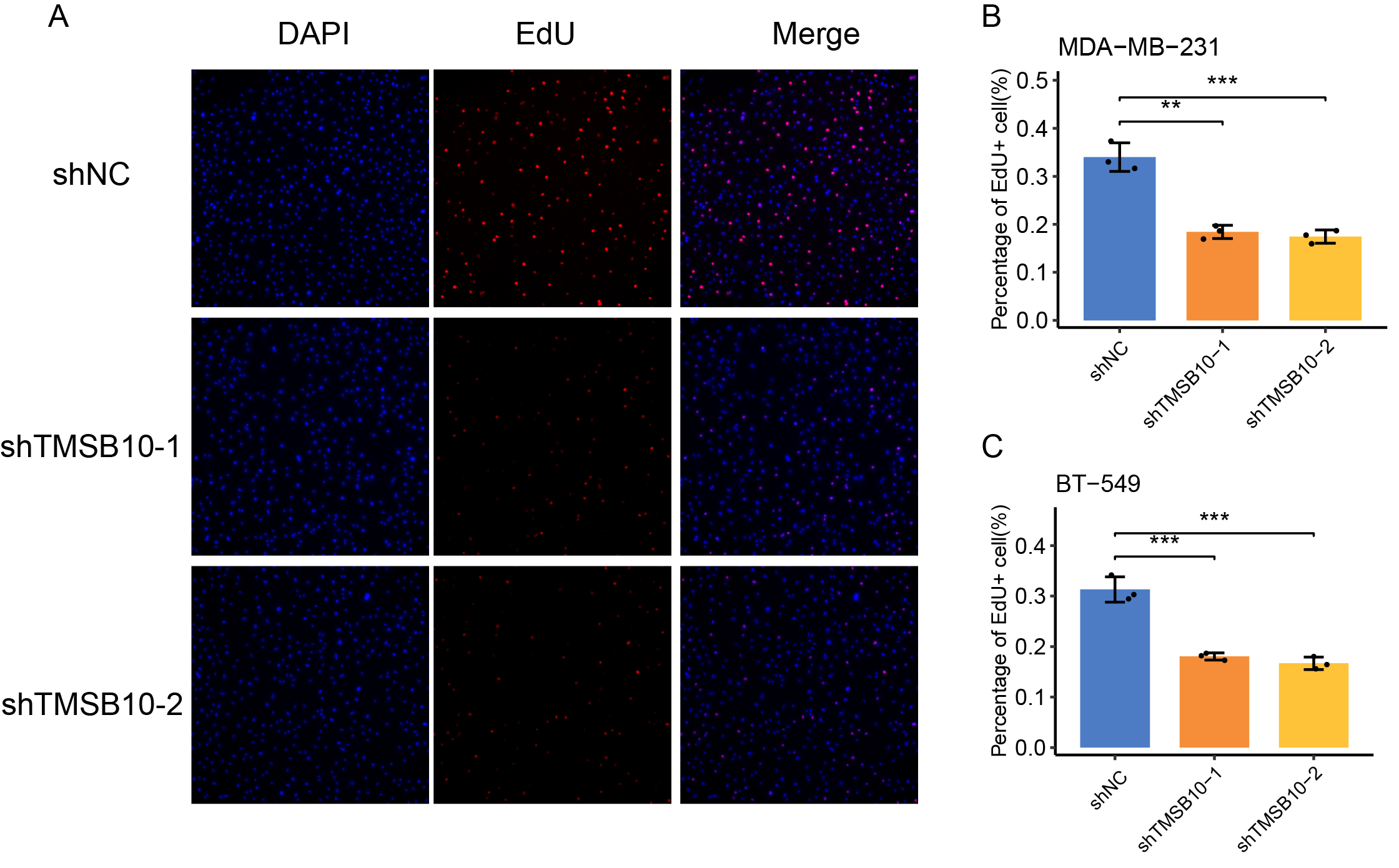


**Figure S5 The cell proliferation assay. A The EdU assay performed on BT-549 cells. B-C** Boxplots showing the percentage of EdU+ cells in both MAD-MB-231 (**B**) and BT-549 (**C**).

**Supplementary Table**

**Table S1. The scRNA-seq samples enrolled in the study**

| Sample | Dataset | treatment | tissue | stage |
| --- | --- | --- | --- | --- |
| GSM5457199 | GSE180286 | untreated | Primary breast cancer | T2N1M0 |
| GSM5457202 | GSE180286 | untreated | Primary breast cancer | T3N2M0 |
| GSM5457205 | GSE180286 | untreated | Primary breast cancer | T1N1M0 |
| GSM5457208 | GSE180286 | untreated | Primary breast cancer | T2N2M0 |
| GSM5457211 | GSE180286 | untreated | Primary breast cancer | T2N1M0 |
| GSM5354513 | GSE176078 | / | Primary breast cancer | / |
| GSM5354514 | GSE176078 | / | Primary breast cancer | / |
| GSM5354515 | GSE176078 | / | Primary breast cancer | / |
| GSM5354516 | GSE176078 | / | Primary breast cancer | / |
| GSM5354517 | GSE176078 | / | Primary breast cancer | / |
| GSM5354518 | GSE176078 | / | Primary breast cancer | / |
| GSM5354519 | GSE176078 | / | Primary breast cancer | / |
| GSM5354520 | GSE176078 | / | Primary breast cancer | / |
| GSM5354521 | GSE176078 | / | Primary breast cancer | / |
| GSM5354522 | GSE176078 | / | Primary breast cancer | / |
| GSM5354523 | GSE176078 | / | Primary breast cancer | / |
| GSM5354524 | GSE176078 | / | Primary breast cancer | / |
| GSM5354525 | GSE176078 | / | Primary breast cancer | / |
| GSM5354526 | GSE176078 | / | Primary breast cancer | / |
| GSM5354527 | GSE176078 | / | Primary breast cancer | / |
| GSM5354528 | GSE176078 | / | Primary breast cancer | / |
| GSM5354529 | GSE176078 | / | Primary breast cancer | / |
| GSM5354530 | GSE176078 | / | Primary breast cancer | / |
| GSM5354531 | GSE176078 | / | Primary breast cancer | / |
| GSM5354532 | GSE176078 | / | Primary breast cancer | / |
| GSM5354533 | GSE176078 | / | Primary breast cancer | / |
| GSM5354534 | GSE176078 | / | Primary breast cancer | / |
| GSM5354535 | GSE176078 | / | Primary breast cancer | / |
| GSM5354536 | GSE176078 | / | Primary breast cancer | / |
| GSM5354537 | GSE176078 | / | Primary breast cancer | / |
| GSM5354538 | GSE176078 | / | Primary breast cancer | / |

**Table S2. The other datasets analyzed in this study**

| Dataset | Description | Type | OS data |
| --- | --- | --- | --- |
| GSE203612 | 3 BRCA samples | Spatial transcriptome | / |
| GSE20685 | 327 BRCA samples | Expression profiling by array | Yes |
| GSE42568 | 104 BRCA samples | Expression profiling by array | Yes |
| GSE58812 | 107 BRCA samples | Expression profiling by array | Yes |
| TCGA-BRCA | 1073 BRCA samples | bulk RNA, CNV, SNV,DNA methylation 450K,MRI | Yes |
| METABRIC | 1250 BRCA samples with treatment information | Expression profiling by array | Yes |
| GSE173839 | 71 BRCA samples received durvalumab/olaparib | Expression profiling by array | Yes |

**Table S3 oligonucleotides used for shRNA**

| Name | Target Sequence (5’ to 3’) |
| --- | --- |
| shTMSB10-1 | CCCAGTCGTGATGTGGAGGAA |
| shTMSB10-2 | CTGCCGACCAAAGAGACCATT |

**Table S4 The primers of TMSB10 and GAPDH**

| Name | Sequence (5’ to 3’) |
| --- | --- |
| TMSB10-F | GAAATCGCCAGCTTCGATAAGG |
| TMSB10-R | TCAATGGTCTCTTTGGTCGGC |
| GAPDH-F | ACAACTTTGGTATCGTGGAAGG |
| GAPDH-R | GCCATCACGCCACAGTTTC |

**Table S5 The correlation between the infiltration level of C1 cluster and clinical characteristics**

| Variables | Total (n = 818) | high (n = 418) | low (n = 400) | p |
| --- | --- | --- | --- | --- |
| Age, Median (Q1,Q3) | 56 (48, 65) | 56 (48, 64) | 56.5 (47, 65) | 0.764 |
| Sex, n (%) |  |  |  | 1 |
| Female | 808 (99) | 413 (99) | 395 (99) |  |
| Male | 10 (1) | 5 (1) | 5 (1) |  |
| Race, n (%) |  |  |  | 0.603 |
| AMERICAN INDIAN OR ALASKA NATIVE | 1 (0) | 0 (0) | 1 (0) |  |
| ASIAN | 55 (7) | 29 (7) | 26 (6) |  |
| BLACK OR AFRICAN AMERICAN | 120 (15) | 66 (16) | 54 (14) |  |
| WHITE | 642 (78) | 323 (77) | 319 (80) |  |
| N_stage, n (%) |  |  |  | 0.726 |
| N0 | 408 (50) | 215 (51) | 193 (48) |  |
| N1 | 272 (33) | 132 (32) | 140 (35) |  |
| N2 | 95 (12) | 50 (12) | 45 (11) |  |
| N3 | 43 (5) | 21 (5) | 22 (6) |  |
| M_stage, n (%) |  |  |  | 0.852 |
| M0 | 804 (98) | 410 (98) | 394 (98) |  |
| M1 | 14 (2) | 8 (2) | 6 (2) |  |
| T_stage, n (%) |  |  |  | 0.014 |
| T1 | 224 (27) | 100 (24) | 124 (31) |  |
| T2 | 476 (58) | 266 (64) | 210 (52) |  |
| T3 | 93 (11) | 42 (10) | 51 (13) |  |
| T4 | 25 (3) | 10 (2) | 15 (4) |  |
| Tumor_Stage, n (%) | |  |  | 0.114 |
| I | 155 (19) | 69 (17) | 86 (22) |  |
| II | 472 (58) | 257 (61) | 215 (54) |  |
| III | 177 (22) | 84 (20) | 93 (23) |  |
| IV | 14 (2) | 8 (2) | 6 (2) |  |

**Table S6 The correlation between the absolute score of C1 cluster and radiomic features**

| v1 | v2 | cor | pvalue |
| --- | --- | --- | --- |
| abosulte.score | original.shape.Maximum2DDiameterSlice | 0.233431407 | 0.025955846 |
| abosulte.score | wavelet-LLH.firstorder.10Percentile | -0.231475729 | 0.027265961 |
| abosulte.score | wavelet-LLH.firstorder.Energy | 0.302412665 | 0.003574031 |
| abosulte.score | wavelet-LLH.firstorder.InterquartileRange | 0.298100334 | 0.00410549 |
| abosulte.score | wavelet-LLH.firstorder.Maximum | 0.218142277 | 0.037777073 |
| abosulte.score | wavelet-LLH.firstorder.MeanAbsoluteDeviation | 0.275870366 | 0.00812505 |
| abosulte.score | wavelet-LLH.firstorder.Minimum | -0.306945476 | 0.003082525 |
| abosulte.score | wavelet-LLH.firstorder.Range | 0.2884433 | 0.005558835 |
| abosulte.score | wavelet-LLH.firstorder.RobustMeanAbsoluteDeviation | 0.283274197 | 0.006510858 |
| abosulte.score | wavelet-LLH.firstorder.RootMeanSquared | 0.220315844 | 0.035862362 |
| abosulte.score | wavelet-LLH.firstorder.TotalEnergy | 0.301076088 | 0.003731766 |
| abosulte.score | wavelet-LLH.firstorder.Variance | 0.346255427 | 0.00077368 |
| abosulte.score | wavelet-LLH.glcm.Autocorrelation | 0.468359627 | 2.84E-06 |
| abosulte.score | wavelet-LLH.glcm.ClusterProminence | 0.439602347 | 1.30E-05 |
| abosulte.score | wavelet-LLH.glcm.ClusterShade | -0.455854708 | 5.61E-06 |
| abosulte.score | wavelet-LLH.glcm.ClusterTendency | 0.318558792 | 0.002087845 |
| abosulte.score | wavelet-LLH.glcm.Contrast | 0.412579747 | 4.83E-05 |
| abosulte.score | wavelet-LLH.glcm.DifferenceAverage | 0.329329197 | 0.001434642 |
| abosulte.score | wavelet-LLH.glcm.DifferenceVariance | 0.344922031 | 0.000813272 |
| abosulte.score | wavelet-LLH.glcm.JointAverage | 0.341452128 | 0.000925114 |
| abosulte.score | wavelet-LLH.glcm.SumAverage | 0.341452128 | 0.000925114 |
| abosulte.score | wavelet-LLH.glcm.SumSquares | 0.388525253 | 0.000141398 |
| abosulte.score | wavelet-LLH.gldm.GrayLevelVariance | 0.352469124 | 0.000611363 |
| abosulte.score | wavelet-LLH.gldm.HighGrayLevelEmphasis | 0.479452525 | 1.52E-06 |
| abosulte.score | wavelet-LLH.gldm.LargeDependenceHighGrayLevelEmphasis | 0.491946435 | 7.33E-07 |
| abosulte.score | wavelet-LLH.gldm.SmallDependenceHighGrayLevelEmphasis | 0.472696461 | 2.23E-06 |
| abosulte.score | wavelet-LLH.glrlm.GrayLevelVariance | 0.352408096 | 0.000612793 |
| abosulte.score | wavelet-LLH.glrlm.HighGrayLevelRunEmphasis | 0.47925013 | 1.54E-06 |
| abosulte.score | wavelet-LLH.glrlm.LongRunHighGrayLevelEmphasis | 0.480183043 | 1.46E-06 |
| abosulte.score | wavelet-LLH.glrlm.ShortRunHighGrayLevelEmphasis | 0.479007684 | 1.56E-06 |
| abosulte.score | wavelet-LLH.glszm.GrayLevelVariance | 0.351707495 | 0.000629428 |
| abosulte.score | wavelet-LLH.glszm.HighGrayLevelZoneEmphasis | 0.476814997 | 1.77E-06 |
| abosulte.score | wavelet-LLH.glszm.LargeAreaHighGrayLevelEmphasis | 0.486306452 | 1.02E-06 |
| abosulte.score | wavelet-LLH.glszm.SmallAreaHighGrayLevelEmphasis | 0.473184381 | 2.17E-06 |
| abosulte.score | wavelet-LLH.ngtdm.Complexity | 0.361988412 | 0.000422238 |
| abosulte.score | wavelet-LLH.ngtdm.Strength | 0.213722366 | 0.041934925 |
| abosulte.score | wavelet-LHH.firstorder.Mean | -0.218469086 | 0.037483838 |
| abosulte.score | wavelet-LHH.glcm.ClusterShade | 0.218382538 | 0.037561309 |

**Table S7 The variables and coefficients of the radiomic model**

| feature | coe |
| --- | --- |
| original.shape.Maximum2DDiameterSlice | 0.04995329 |
| wavelet.LLH.glcm.ClusterShade | -0.2143939 |
| wavelet.LLH.gldm.LargeDependenceHighGrayLevelEmphasis | 0.3070862 |
| wavelet.LLH.ngtdm.Strength | -0.02903903 |
| wavelet.LHH.firstorder.Mean | -0.04715325 |

**Table S8. Univariate COX regression analysis of candidate genes in the training set (GSE20685)**

| Gene | HR | z | pvalue | lower95 | upper95 |
| --- | --- | --- | --- | --- | --- |
| S100A9 | 1.14359887 | 2.993430063 | 0.002758607 | 1.047414776 | 1.248615549 |
| PGK1 | 1.937883051 | 2.945800182 | 0.003221204 | 1.247833172 | 3.009529481 |
| PPIA | 5.98974956 | 2.795466818 | 0.005182482 | 1.707458483 | 21.01198954 |
| S100A8 | 1.109962641 | 2.591583994 | 0.009553522 | 1.025752394 | 1.20108622 |
| PERP | 1.437527568 | 2.463076016 | 0.013775071 | 1.076948524 | 1.91883406 |
| ATG5 | 1.545546347 | 2.390654578 | 0.016818368 | 1.081593949 | 2.208512272 |
| P4HB | 1.773312825 | 2.127689007 | 0.033362877 | 1.046192657 | 3.005792819 |
| KLF6 | 1.523563821 | 2.102940553 | 0.035470966 | 1.029040554 | 2.255738812 |
| CFL1 | 2.762931419 | 2.029549306 | 0.042402372 | 1.035458882 | 7.372373895 |
| CD24 | 1.304333639 | 2.008248586 | 0.044616882 | 1.006408524 | 1.690452932 |
| TMSB10 | 2.214984104 | 1.996334078 | 0.045897571 | 1.014593589 | 4.835586024 |
